# Multi-omics analysis defines 5-fluorouracil drug resistance in 3D HeLa carcinoma cell model

**DOI:** 10.1186/s40643-021-00486-z

**Published:** 2021-12-23

**Authors:** Lin Wang, Xueting Wang, Tong Wang, Yingping Zhuang, Guan Wang

**Affiliations:** 1 State Key Laboratory of Bioreactor Engineering, East China University of Science and Technology, Shanghai, People’s Republic of China; 2 Qingdao Innovation Institute of East China University of Science and Technology, Shanghai, People’s Republic of China

**Keywords:** Drug resistance mechanism, HeLa carcinoma cells, Multicellular tumor spheroid, Multi-omics analysis, Preclinical evaluation, Tumor metabolism

## Abstract

Cervical cancer is a serious health problem in women around the globe. However, the use of clinical drug is seriously dampened by the development of drug resistance. Efficient in vitro tumor model is essential to improve the efficiency of drug screening and the accuracy of clinical application. Multicellular tumor spheroids (MTSs) can in a way recapitulates tumor traits in vivo, thereby representing a powerful transitional model between 2D monolayer culture and xenograft. In this study, based on the liquid overlay method, a protocol for rapid generation of the MTSs with uniform size and high reproducibility in a high-throughput manner was established. As expected, the cytotoxicity results showed that there was enhanced 5-fluorouracil (5-FU) resistance of HeLa carcinoma cells in 3D MTSs than 2D monolayer culture with a resistance index of 5.72. In order to obtain a holistic view of the molecular mechanisms that drive 5-FU resistance in 3D HeLa carcinoma cells, a multi-omics study was applied to discover hidden biological regularities. It was observed that in the 3D MTSs mitochondrial function-related proteins and the metabolites of the tricarboxylic acid cycle (TCA cycle) were significantly decreased, and the cellular metabolism was shifted towards glycolysis. The differences in the protein synthesis, processing, and transportation between 2D monolayer cultures and 3D MTSs were significant, mainly in the heat shock protein family, with the up-regulation of protein folding function in endoplasmic reticulum (ER), which promoted the maintenance of ER homeostasis in the 3D MTSs. In addition, at the transcript and protein level, the expression of extracellular matrix (ECM) proteins (e.g., laminin and collagen) were up-regulated in the 3D MTSs, which enhanced the physical barrier of drug penetration. Summarizing, this study formulates a rapid, scalable and reproducible in vitro model of 3D MTS for drug screening purposes, and the findings establish a critical role of glycolytic metabolism, ER hemostasis and ECM proteins expression profiling in tumor chemoresistance of HeLa carcinoma cells towards 5-FU.

**Graphical Abstract:**

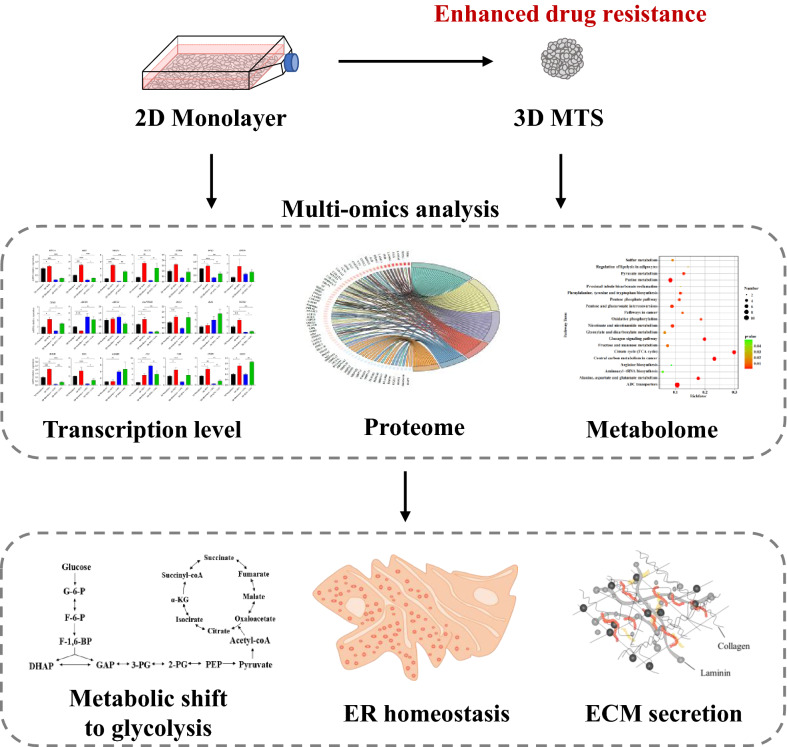

**Supplementary Information:**

The online version contains supplementary material available at 10.1186/s40643-021-00486-z.

## Introduction

Cancer is the leading cause of death among people under the age of 70 in most countries. According to the statistics from the International Agency for Research on Cancer (IARC), there were 19.29 million new cancer cases worldwide in 2020, resulting in the death of 9.96 million patients (Wild et al. 2020). Tumorigenesis is broadly dependent on the reprogramming of cellular metabolism as both direct and indirect consequence of oncogenic mutations. To satisfy rapid growth and proliferation, tumor cells require a large amount of nutrients to meet their material and energy needs (Zhu and Thompson 2019). Tumor cells mainly rely on aerobic glycolysis (also known as the Warburg effect) for rapid energy and precursor supply, which is accompanied by the production of large amounts of lactate (Vander Heiden et al. 2009). Apart from the glucose as a bioenergetic substrate, the conversion of glutamine to α-ketoglutarate and subsequent entering the tricarboxylic acid cycle (TCA cycle) is often necessary for tumor cell growth (Fan et al. 2013; Son et al. 2013). Also, recent findings have showed that accumulation and recycling of lactate and ammonium can contribute to the progression and occurrence of cancer (Martínez‐Monge et al. 2018; Spinelli et al. 2017). In addition, the nucleotide metabolic pathway of tumor cells is highly efficient and nucleotide imbalance further induces tumor-associated mutations (Aird and Zhang 2015). Based on the complex genetic diversities and epigenetic differences of tumor cells, there are great differences between the individual differences of different patients and the tissue heterogeneity of the same patient are huge, which seriously hinders the effective treatment of tumors (Hensley et al. 2016; Singh et al. 2010).

Cervical cancer is the second leading cause of deaths in female cancer patients of childbearing age, and there were 600 thousand new cases and 340 thousand deaths worldwide in 2020 (Sengupta and Honey 2020). At present, surgery, radiotherapy and chemotherapy are the most commonly used methods for the treatment of cervical cancer; however, surgery cannot remove all cancer cells and the metastasis of cancer cells exacerbates the difficulty of treatment, while radiotherapy and chemotherapy have serious side effects and induce drug resistance (Keshavarz-Fathi and Rezaei 2021). For example, 5-fluorouracil (5-FU), an uracil analog, is a commonly used clinical drug for cancer treatment, which targets thymidylate synthase (TYMS) to affect the de novo nucleotide synthesis pathway (Longley et al. 2003). Although 5-FU has a good therapeutic effect for cancer patients in the early stage, the evolution of chemoresistance towards antitumor drug hampers its clinical use. For example, Giacchetti et al. showed that the overall response rate of 5-FU for patients with advanced colon cancer was only 10–15% (Giacchetti et al. 2000).

Apart from the drug resistance, the lack of preclinical model further aggravates the attrition rate in the clinical drug development. It has been estimated that the drug attrition rates are as high as 95% tested in phase I clinical trials; 67% and 33% of all drugs that enter Phase II and Phase III clinical trials fail to transit into the next stage, respectively (Santo et al. 2017). Since the 1950s, monolayer cells have been used for in vitro drug screening due to its simple, cheap and repeatable features (Hickman et al. 2014). However, monolayer cells cannot completely reproduce the physiological and pathological microenvironment of tumor cells in vivo, and thus could not accurately characterize the function and phenotype of tumor cells in vivo (Sarvestani et al. 2020). The use of preclinical models that are unable to fully recapitulate the complexity of tumors would seriously affect the transition of new anticancer treatment to the clinic. The traditional patient-derived tumor xenograft (PDX) model could better retain the tumor tissue characteristics of patients, but with high cost and long periods (Jiang et al. 2021). Therefore, the construction of efficient in vitro tumor model is of great significance to improve the efficiency of drug screening and the accuracy of clinical application. In 1970, Sutherland and colleagues first proposed the 3D multicellular tumor spheroid (MTS), and pointed out that the physiological characteristics of MTSs were similar to the avascular tumor nodules, tumor micro-metastasis and intravascular areas of tumor (Inch et al. 1970). Compared with the 2D monolayer culture, MTSs behave closer microenvironment to tumors in vivo, which could more accurately characterize the function and phenotype of tumor tissues, with closer gene expression profiles, protein expression profiles, and metabolite profiles to the counterparts of the PDX model (Lauschke et al. 2019; Wang et al. 2020; Zietarska et al. 2007). Numerous reports have shown that MTSs were more resistant to radiotherapy and chemotherapy, and even the aggregates of 25–50 tumor cells also have shown enhanced resistance than 2D monolayer cells (Yu et al. 2010).

Multi-omics analysis of the effect of antitumor drug on tumor cell metabolism renders a more comprehensive exploration of the mechanism of drug resistance (Guang et al. 2018). Systems biology research has contributed to clinical drug development, judgment of patient prognosis and personalized treatment (Magani et al. 2018; Veenstra 2021). Genomics, transcriptomics, proteomics and metabolomics are most powerful systems biology tools, which have been used in combination or alone to elucidate the mechanism of tumor resistance. For example, based on transcriptome and proteomic techniques, Lu et al. found that the inhibition of STAT3 signal transduction contributed to inhibiting EMT and down-regulating the expression of stem genes, thereby inhibiting tumor progression, metastasis and chemical resistance (Lu et al. 2019). Nonetheless, to the best of our knowledge, numerous studies were carried out with 2D monolayer cultures, while multi-omics studies are rarely performed with 3D in vitro tumor models (Kalfe et al. 2015; Schroll et al. 2017; Seker et al. 2019).

Although MTSs are capable of accurately describing the effects of drugs in vivo, the use of 3D model is still limited due to the limits in preparation and analysis methods, and only less than 30% of researchers used 3D model for anticancer drug screening, evaluation and other relevant studies (Hutmacher 2010). Therefore, in order to improve the predictability and availability for screening anticancer drug candidates for preclinical trials, in the present study we established a rapid, reproducible and standardized MTSs culture method. Furthermore, to investigate the phenotypic differences of HeLa carcinoma cells with the 5-FU treatment under both 2D monolayer cultures and 3D MTS, we leveraged multi-omics analysis across transcript, protein and metabolite levels to better understand the key regulatory genes and related metabolic pathways responsible for the drug resistance mechanism.

## Materials and methods

### Cell lines and media

Cell lines HeLa (carcinoma cells), HCT116 (colon cancer cells), HepG2 (liver cancer cells), A549 (lung cancer cells), MCF-7 (breast cancer cells), 5637 (bladder cancer cell) and L-02 (human hepatocytes) were purchased from American Type Culture Collection (ATCC, Manassas, VA, USA). UCF (umbilical cord fibroblast) was obtained from National Center of Bio-Engineering & Technology (Shanghai, CN). PBMC (peripheral blood mononuclear cells) was obtained from Otwo Biotech (Shenzhen, CN).

All cells were cultivated in Dulbecco’s modified Eagle medium (DMEM, 11995-06, Gibco, Grand Island, NY, USA) containing 10% fetal bovine serum (FBS, 10099-141, Gibco, Grand Island, NY, USA), 100 IU/mL penicillin and 100 mg/mL streptomycin (B540732, Sangon Biotech, Shanghai, CN). All cultures were maintained in an incubator (Thermo Fisher Scientific, Waltham, MA, USA) at 37 °C with 5% CO_2_ and 95% humidity, and passaged when the confluence of cells reached 80%–90%.

### Screen for optimized conditions to generate single tumor spheroid

**Fig. 1:**
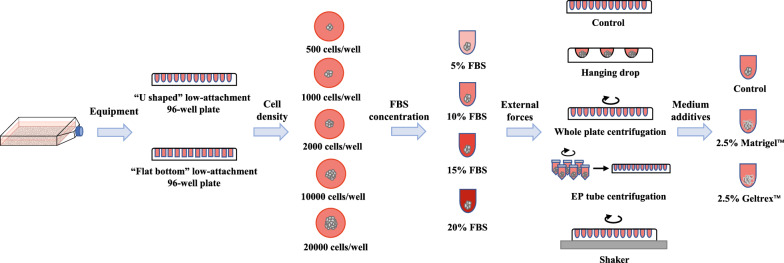
Schematic illustration of the generation of single tumor spheroid

To establish a protocol for rapid spheroid generation in a high-throughput manner, the formation of single tumor spheroid per well in a 96-well plate was fully optimized (Fig. 1). Cells grown as a monolayer were detached with trypsin to generate a single-cell suspension. The cell number was determined using Countstar (ALIT Life Science, Shanghai, CN) with the automatic cell counter software. To prevent cell attachment, Ultra-low attachment 96-well plates in both “Flat bottom” (3474, Corning, NY, USA) and “U-shaped” (7007, Corning, NY, USA) were utilized. For optimal spheroid formation, the cell seeding density (500–20,000 cells/well), serum concentrations (5%–20%), external forces (gravity and centrifugal forces), and medium additives (2.5% of Geltrex™ (A15696-01, Gibco, Grand Island, NY, USA) and Matrigel™ (356,237, Corning, NY, USA) were tested. For external forces, the experiments were carried out as follows: 1. Control: 200 μL cell suspension was directly seeded without treatment; 2. hanging drop method: seeded 20 μL cell suspension at the bottom of the well plate, turned the culture plate upside down and incubated for 48 h, then put the well plate upright and supplemented with 180 μL medium; 3. centrifugation of the whole plate: seeded 200 μL cell suspension, and the whole culture plate was centrifuged at 1000 g for 10 min; 4. EP tube centrifugation: added 200 μL cell suspension to a 1.5 mL EP tube, centrifuged at 1000 g for 10 min, and then transferred the cell clusters to a 96-well plate; 5. rotary shaker: seeded 200 μL cell suspension, and placed the whole plate on a shaker at 60 RPM.

Through the screening of MTSs culture conditions, we have established a rapid MTS formation protocol to produce MTSs. In brief, the cell suspension was diluted to 10^4^ cells/mL, which was fully mixed with 2.5% (v/v) Matrigel™, and then a volume of 200 μL of this cell suspension was added to each well of "U-shaped" low-attachment 96-well plates. The spheroid formation was initiated by centrifuging the plate at 1000 g for 10 min. Afterwards, the plates were cultured in an incubator at 37 °C with 5% CO_2_ and 95% humidity for 6 days. In this study, the protocol was exactly followed in the formation of multicellular tumor spheroids, unless otherwise specified.

### Determination of spheroid growth kinetics

During the spheroid culture process, the growth of MTSs was assessed and recorded using electron microscopy (Life Technology, Carlsbad, CA, USA) every 24 h, and the diameter and roundness of MTSs were calculated by Image Pro Plus 6.0. To determine the active cell number in the spheroid, 3 cell spheroids were taken every 24 h, the supernatant discarded, and then digested with Accutase™ cell digestive solution (40506ES60, Yesen, Shanghai, CN) at 37 ℃ for 30 min to form single-cell suspension. The suspension was stained by trypan blue and then counted using Countstar.

### Scanning electron microscopy

The morphology and structure were observed and recorded by scanning electron microscope (SEM, S-3400 N, Hitachi, Tokyo, JP). The MTSs were collected and washed with PBS twice, then fixed with 2.5% glutaraldehyde in deionized water for 1 h and washed with deionized water for 5 times. The fixed spheroids were subsequently dehydrated with a graded ethanol series (25%, 50%, 75%, 95% and 100%) for 1 h, respectively, then washed with tert-butanol for 30 min (three times) and freeze-dried at − 80 ℃ overnight. The freeze-dried MTSs were fixed on the sample post with double-sided carbon conductive tape, and coated with palladium alloy to make the sample have good conductivity for the SEM analysis.

### Cell viability

Single cell suspensions from both 2D monolayer culture and 3D MTSs were prepared as described above. About 10^6^ cells were collected for cell cycle and apoptosis analysis by cell cycle analysis kit (C1052, Beyotime, Shanghai, CN) and apoptosis analysis kit (40302ES60, Yeasen, Shanghai, CN), respectively. The operation steps were detailed in the kit instructions. The flow cytometer (Beckman Coulter, Brea, CA, USA) was used for detection, and the obtained data were analyzed by FlowJo 10.5.

### Cytotoxicity assay

The cytotoxicity of 5-FU to tumor cells cultured under both 2D monolayer and 3D MTSs was determined. 2D monolayer culture: HeLa cells (10,000 cells/well) were seeded in the 96-well plates and incubated for 24 h; 3D MTS: HeLa cells (2000 cells/well) were seeded in the 96-well plates and cultured for 6 days. Then removed the medium and incubated with different concentrations of 5-FU (MS0249, Mkbio, Shanghai, CN) for 48 h. Then removed the medium and added 200 μL CCK-8 staining solution (40203ES92, Yeasen, Shanghai, CN) diluted with 1:10 medium to each well. After incubation at 37 °C for 2 h, the absorbance of each well was determined at λ = 450 nm by fluorescence microplate reader (Thermo Fisher Scientific, Waltham, MA, USA). Cellular viability was calculated according to the following equation. Half inhibitory concentration (IC_50_) was calculated using GraphPad Prism 8.

$$\text{Cellular viability}=\frac{{A}_{450, \mathrm{ dose}}-{A}_{450, \mathrm{blank}}}{{A}_{450, 0\mathrm{ dose}}-{A}_{450, \mathrm{blank}}}\times 100\%.$$

### Mitochondrial respiratory capacity assays

Tumor cells from both 2D and 3D MTSs were digested to prepare single-cell suspensions. Oxygraph-2 k mitochondrial function analyzer (Oroboros, Innsbruck, Austria) was used to measure the cellular oxygen consumption rate (OCR) under the following four conditions: routine, normal cell respiration level; leak, ATP synthase was inhibited by 1 μL 4 mg/ml oligomycin (Sigma-Aldrich, San Luis, MO, USA). Electron transfer system (ETS): the oxygen consumption level reached the maximum, with supplement of 1 μL 1 mM FCCP (Carbonylcyanide p-trifluoromethoxyphenylhydrazone, Abcam, Cambridge, UK) to destroy the proton gradient and mitochondrial membrane potential. Residual oxygen consumption (ROX): the residual intracellular oxygen consumption was measured by adding 1 μL 1 mM rotenone (Abcam, Cambridge, UK) and 1 μL 1 mM antimycin A (Abcam, Cambridge, UK) to inhibit mitochondrial complex I/III and mitochondrial respiration. Basal respiration: the OCR difference value between routine and ROX. ATP production: the OCR difference value between routine and leak. Maximal respiration: the OCR difference value between ETS and ROX. Spare respiratory capacity: the OCR difference value between ETS and routine. Non-mitochondrial respiration: the OCR value of ROX.

### Reaction oxygen species (ROS) measurement

The ROS production capacity of cells cultured under both 2D monolayer and 3D MTSs was determined using the ROS detection kit (50101ES01, Yeasen, Shanghai, CN), according to the manufacturer’s protocol. The fluorescence dye DCFH-DA (2,7-dichlorodi-hydrofluorescein diacetate), without its own fluorescence, could freely shuttle through the cell membrane, and is hydrolyzed by intracellular esterase to generate DCFH, which could not shuttle through the cell membrane, and accumulates in cells. Non-fluorescent DCFH could be oxidized by intracellular ROS to produce DCF with green fluorescence. The fluorescence intensity was measured by fluorescence microplate reader (Thermo Fisher Scientific, Waltham, MA, USA) at the excitation wavelength of 488 nm and the emission wavelength of 525 nm.

### Determination of the concentration of extracellular metabolites

During the cell culture process, the concentration of glucose, glutamine, lactate, and ammonia in the supernatant was determined by a biochemical analyzer (Roche, Basel, CH). Control: 2000 cells were seeded in 96-well plates per well without treatment under both 2D monolayer culture and 3D MTSs. 5-FU treatment: under 2D monolayer culture, 2000 cells were seeded in 96-well plates per well and cultured for 24 h. After that, the supernatant discarded and then cultured with 16 μM 5-FU; under 3D MTSs, 2000 cells were seeded in 96-well plates per well and cultured for 6 days. After that, the supernatant discarded and then cultured with 16 μM 5-FU.

### Transcriptional analysis of tumor resistance-related genes

According to the manufacturer’s protocol, total RNA was extracted using TRIeasy™ Total RNA Extraction Reagent (10606ES60, Yeasen, Shanghai, CN), and RNA concentration and purity were determined by Nano Drop spectrophotometer (Thermo Fisher Scientific, Waltham, MA, USA). The RNA purity was considered as qualified when A_260_/A_280_ was between 1.8 and 2.0. The procedures about reverse transcription (11141ES10, Yeasen, Shanghai, CN) and quantitative polymerase chain reaction (qPCR, 11198ES08, Yeasen, Shanghai, CN) were detailed in the kit instructions. The qPCR reaction was performed by real-time fluorescent quantitative PCR system (Thermo Fisher Scientific, Waltham, MA, USA). The primer sequences involved in this study can be found in Additional file 1: Table S1, and the transcription level of each gene was standardized by the internal control gene, *ACTB*, which is encoding for β-actin.

### Proteome analysis

2D monolayer and 3D MTSs cells treated with and without 5-FU were collected in four groups, with three biological replicates in each group and about 5 × 10^6^ cells in each sample. The proteome was determined using Tandem Mass Tag (TMT) technique. The main processes can be summarized as follows: protein extraction, enzymatic hydrolysis, peptide labeling, LC–MS detection, database analysis and processing. For specific procedures, please refer to Additional file 1. After LC–MS detection and database search, the trusted proteins were screened according to the criteria of Score Sequest HT > 0, unique peptide ≥ 1, and removal of the blank values. Next, the independence of samples was determined by principal component analysis (PCA) using the trusted protein. Based on the trusted proteins, the proteins with *T*-test *p*-value < 0.05 and Fold change > 1.5 were determined as differential proteins. Hierarchical clustering of differential proteins was performed using R (3.6.2) ComplexHeatmap package (2.4.3). Subsequently, the differential protein data were imported into the Gene Ontology (GO) database (http://geneontology.org/) to analyze the biological process, cellular component and molecular function. The chord diagrams of GO enrichment analysis were drawn by GOplot package (1.0.2). Through the KEGG database (https://www.kegg.jp/kegg/pathway.html), pathway enrichment analysis of differential proteins was performed. The bubble charts of KEGG analysis were drawn by ggplot2 package (3.3.2). The STRING database (https://www.string-db.org/) was used to analyze the interactions between proteins, and the top 25 proteins were selected for connectivity as hub proteins by which the network diagram of the hub protein interaction was drawn by Python (3.9.0) networkx package (1.9.1).

### Metabolome analysis

The cells were collected in the same way as the proteome analysis. The main process of non-targeted metabolomics detection includes metabolite extraction, GC–MS analysis, database analysis and processing. For specific procedures, please refer to Additional file 1. After GC–MS detection and database search, the PCA analysis was used to determine the independence of samples. Orthogonal partial least squares-discriminant analysis (OPLS-DA) was used to screen differential metabolites. The screening criteria were VIP value of PC1 > 1 in OPLS-DA and T-test p-value < 0.05. Next, the hierarchical clustering and KEGG analysis of differential metabolites were then performed in the same way as proteomics.

### Statistical analysis

All data in this study were expressed as mean ± SD. Significant analysis was performed by GraphPad Prism 8.0, with *p* < 0.05, *p* < 0.01 and *p* < 0.001 indicated that there are significant (*), highly significant (**) and extremely significant (***) difference between the experimental and control groups.

## Results

### Rapid generation of high-throughput 3D MTSs using an optimized liquid overlay method

To efficiently and economically prepare size-controllable multicellular tumor spheroids, key factors such as cell seeding density, serum concentrations, external forces, and medium additives were systematically optimized (Additional file 1: Table S2). Based on the liquid overlay, cells were seeded on the low-attachment plate coated with non-viscous polymer such that the interaction between cells was greater than the interaction between cells and matrix, and the cells spontaneously gathered into clusters (Costa et al. 2018). The well plates had different shapes, and the effect of tumor cells aggregation in the "U shaped" plates was better than that of the "flat bottom" plates (Additional file 1: Table S2). In the "flat-bottom" plates, the cells in each well gathered into multiple cell clusters with uneven shapes and sizes, while in the "U shaped" plates, the cells in each well could aggregate into a single-cell spheroid each well under the gravity and interaction of the cells.

Due to the oxygen and metabolite concentration gradients caused by mass transfer limitations, MTSs with a diameter over 500 μm could establish three main cell layers, namely the proliferating cell layer on the surface, the resting cell layer in the middle, and the hypoxic necrotic core, which has been reported as one of most important characteristics of MTSs (Hirschhaeuser et al. 2010). The size of MTSs was closely related to the seeding density and the culture time. By recording and observing MTSs every day, we found that the growth of MTSs reached the plateau on the 6th day, on which the MTSs were chosen for the follow-up experiments. The initial seeding density directly determined the final diameter of the MTSs. The diameters of the MTSs on the 6th day were 182.54 μm, 367.21 μm, 512.34 μm, 883.43 μm, 1050.31 μm, with the margin of error no more than 10%, when the seeding densities were 500, 1000, 2000, 10,000 and 20,000 cells/well, respectively (Additional file 1: Table S2).

Serum contains lots of cytokines, collagen, and adhesion factors, which have significant effects on the growth and proliferation of cells (Brown et al. 2019). As shown in Additional file 1: Table S2, the low concentration of FBS was detrimental to the formation of MTSs. Under the condition of 5% FBS, the contact between cells was impaired and thus unable to gather into clusters. Compared with 10% serum, increasing the FBS concentration (15%, 20% FBS) could appropriately improve cell aggregation, but the result was not significant. Considering the cost, 10% FBS was chosen.

The traditional liquid overlay method would promote cells aggregation by introducing external forces to enhance the contact between cells (Costa et al. 2018). Therefore, we investigated the formation and growth of MTSs under the conditions as described in “Materials and methods” section. Compared with the control group, the hanging drop method had poor reproducibility with difficulty to form a single uniform cell spheroid. Besides this, the medium was easy to volatile; EP tube centrifugation significantly improved cell aggregation and reproducibility, which suits lab-scale preparation of 3D MTS for preclinical research, but it was time-consuming and difficult to translate to large-scale production; as for rotary shaker method, the cell clusters were gradually compacted and denser, but it was difficult for the cells to grow and pelletize; by contrast, the whole plate centrifugation significantly improved the roundness and repeatability of MTSs, and it was easy to operate.

To further promote the growth of MTSs, we tested the additives in the culture system. Basement membrane extracts such as Geltrex™ and Matrigel™ contain abundant ECM protein, such as cytokines, laminin, collagen, etc., could promote tumor cell growth, proliferation and invasion, and are usually used in the construction of 3D models for animal cells (Benton et al. 2014). Therefore, we evaluated the effect of 2.5% Geltrex™ and 2.5% Matrigel™ in the formation of MTSs. We found that 2.5% Geltrex™ exerted no significant effect on cell aggregation and growth, while the 2.5% Matrigel™ had extremely obvious promoting effects on the roundness and uniformity of MTSs, and also improved the growth of MTSs (Additional file 1: Table S2).

In summary, through the screening of MTSs culture conditions, we have established a rapid MTS formation protocol that allows to produce uniform and controllable MTSs (the details are described in “Materials and methods” section). In addition to Hela cells used in the follow-up study, other tumor cell lines (colon cancer cells HCT116, liver cancer cells HepG2, breast cancer cells MCF-7, lung cancer cells A549, bladder cancer cells 5637) and normal cell line (human hepatocytes L-02) can form reproducible cell spheroids following this protocol (Additional file 1: Fig. S1). Not only this, this protocol can be extended to co-culturing model with stromal cells (such as fibroblasts UCF, immune cells PBMC) to construct multi-component MTSs (Additional file 1: Fig. S1).

### Physiological characterization of 3D MTSs

**Fig. 2:**
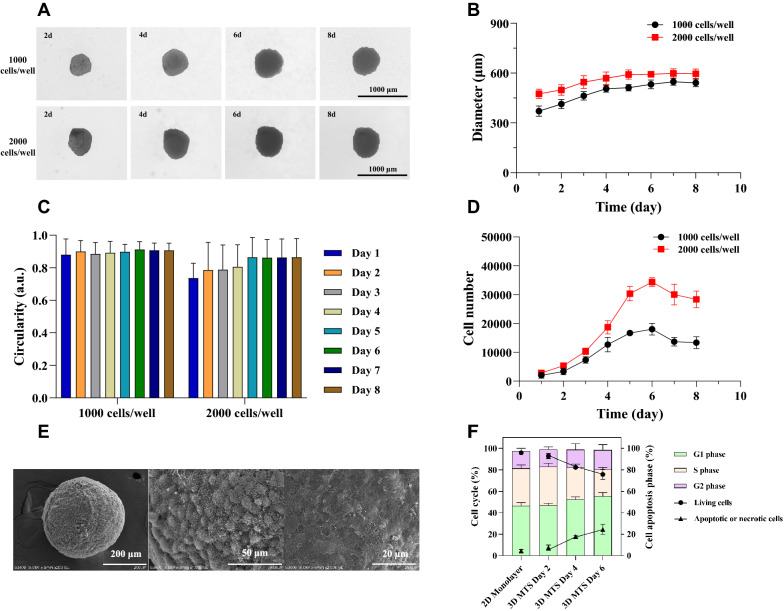
Physiological characterizations of the growth dynamics of 3D MTSs over the culture age. **a** Optical imaging of 3D MTSs. **b** Diameter of 3D MTSs. **c** Circularity of 3D MTSs. **d** Growth curve of 3D MTSs. **e** The morphology and microstructure. **f** Cell apoptosis and cell cycle

To monitor the growth dynamics of MTSs, the diameter, roundness and cell growth of MTSs were recorded and assessed every day. In the initial stage of MTSs formation, cells grew slowly and were easy to distinguish individual cells (Fig. 2a). Driven by the interaction and contact between the cells, cells gradually gathered into clusters. After 2 days of culture, the cells entered the logarithmic growth phase, proliferated rapidly and gradually formed smooth surfaces. On the 6th day, MTSs reached the plateau stage, with the largest diameter of 532.21 ± 21.42 μm and 592.54 ± 13.64 μm, respectively, when the seeding densities were 1000 and 2000 cells/well (Fig. 2b). The roundness of obtained MTSs exceeded 0.9 (Fig. 2c), with the margin of error no more than 10%. The number of living cells in a single MTS reached the maximum on day 6, approx. 17 times the initial amount (Fig. 2d). After that, the boundaries of MTSs began to be wrinkled and blurred, and the spheroids gradually disintegrated.

The morphology and microstructure of MTSs cultured for 6 days were observed by the SEM, and the MTSs showed a good 3D structure and regular spherical shape (Fig. 2e, Additional file 1: Fig. S2). Interestingly, MTSs of different cell types had different surface structures. The surface of the single-component HeLa cell spheroid was relatively smooth (Fig. 2e), while the multi-component MTSs retained the more obvious tissue structures (Additional file 1: Fig. S2). When co-cultured with fibroblasts such as UCF, the fibroblasts extended outwards MTSs. Obvious vesicles were observed in the MTSs co-cultured with immune cells such as PBMC. This indicates that the more complex 3D model would preserve more completely biological characteristics of tumor tissues in vivo.

Compared with 2D monolayer cells, the cell proliferation rate in 3D MTSs was reduced. The specific growth rate (µ) of cells cultured for 2 days under 2D conditions was 0.0455 h^−1^, while the µ of cells in MTSs cultured for 2, 4, and 6 days were 0.0263 h^−1^, 0.0246 h^−1^, 0.0052 h^−1^, respectively, with the margin of error no more than 13.6% (Additional file 1: Fig. S3). The cellular viability of 3D MTSs cultured for 2 days is similar to that of 2D monolayer cells; however, the percentage of living cells and apoptotic/necrotic cells in 3D MTSs starts to decrease and increase, respectively, along with the culture time (Fig. 2f). Compared with 2D monolayer culture, the cell cycle in 3D MTSs was blocked throughout the cultivation. In addition, cells in the 3D MTS are accumulated and decreased over the culture age in the G1 and S-phase, respectively (Fig. 2f). However, there was no significant change in the proportion of cells in G2 phase between 2D monolayer culture and 3D MTSs.

### Chemosensitivity of HeLa tumor spheroids following 5-FU treatment

**Fig. 3:**
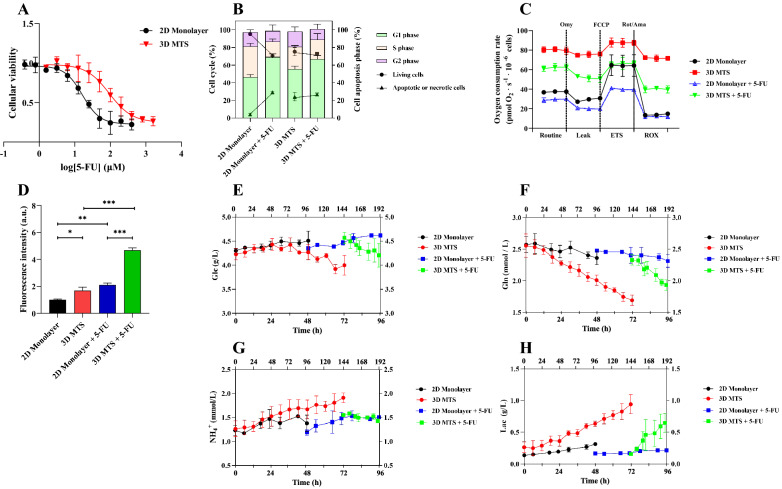
The effect of 5-FU on 3D MTSs. **a** Cytotoxicity assay. **b** Cell apoptosis and cell cycle. **c** Mitochondrial respiration profile. **d** ROS measurement. The measured concentrations of extracellular **e** glucose, **f** glutamine, **g** ammonium and **h** lactate. Omy, oligomycin; Rot, rotenone; Ama, antimycin A. The culture time of 2D monolayer culture and 3D MTS was shown on the bottom and top axis, respectively

The cytotoxicity of 5-FU to HeLa cells cultured under both 2D monolayer and 3D MTSs was evaluated (Fig. 3a). 3D MTSs showed stronger resistance than 2D monolayer cells, and after the 5-FU treatment for 48 h the IC_50_ of MTSs (93.88 μM, 95% confidence interval: 64.88–148.5 μM) was approximately 5.72 times that of 2D monolayer culture (16.42 μM, 95% confidence interval: 12.73–21.14 μM). Therefore, we treated the HeLa carcinoma cells from both 2D and 3D models with 16 μM 5-FU for 48 h in the following study. Next, the changes of cell apoptosis and cell cycle were compared before and after the 5-FU treatment (Fig. 3b). The effect of 5-FU on cells in 3D MTSs was significantly reduced compared with 2D monolayer culture. After the 5-FU treatment, the percentage of living cells and apoptotic cells was decreased and increased, respectively, in 2D monolayer, while there were no significant changes in 3D MTSs. To our knowledge, 5-FU mainly targets S-phase cells (Ijichi et al. 2014). After the 5-FU treatment, the cell cycle was blocked in G1 phase in both 2D monolayer and 3D MTSs. Meanwhile, we observed a decrease in the proportion of S-phase cells and a constant proportion of G2 phase cells in the 2D monolayer after the 5-FU treatment, while no significant changes were observed in the 3D MTS.

To adapt to rapid metabolic requirements of tumor cells, the metabolic pathways have significantly changed to metabolize nutrients in a manner conducive to proliferation rather than efficient ATP production (Seyfried et al. 2020). Mitochondrion is the energy factory and the main position of oxygen consumption, and the abnormal mitochondrial metabolism in tumor cells are often related to drug resistance (Yan and Li 2018). According to the mitochondrial respiration profile (Fig. 3c), the oxygen consumption in 3D MTSs was much higher than that of 2D monolayer cells, which was mainly due to the significant increase of non-mitochondrial respiration. The results showed that the non-mitochondrial respiration of MTSs was about 4.60 and 3.19 times that of 2D cultures under the control and 5-FU treatment conditions, respectively. The possible reason for the increased non-mitochondrial respiration was that the main energy source shifted from mitochondrial oxidative phosphorylation to glycolysis. Contrary to this, the spare respiratory capacity of MTSs was reduced by 74.98% and 63.31% relative to the 2D culture, which may be associated with mitochondrial dysfunction. Furthermore, the mitochondrial basal respiration and maximum respiration capacity were reduced by 68.56% and 71.81% in 3D MTSs relative to the 2D culture under the control conditions, while the ATP synthesis capacity was almost constant. After the 5-FU treatment, the mitochondrial basal respiration and maximum respiration capacity were almost constant, while the ATP synthesis capacity was about 62.40% reduced in 3D MTSs as compared with the 2D cultures. The further inhibition of 5-FU on the mitochondrial ATP production capacity of 3D MTS aggravated the dependence of MTS on the glycolytic pathway. As the major hub of cellular energy generation, mitochondrion, is also the main source of reactive oxygen species (ROS) and it has been reported that the ROS level can be reduced if glycolysis as the main energy source (Herst et al. 2004). However, we found that the ROS production capacity in 3D MTSs was significantly higher than 2D monolayer cells (Fig. 3d). This may be ascribed to the up-regulation of the non-mitochondrial oxidative system, which is accompanied by the increase of oxygen consumption.

As can be seen from Fig. 3e, f, in the presence of glucose and glutamine, tumor cells preferred to use glutamine. Under control conditions, cells cultured in 2D monolayer cultures and 3D MTSs mainly used glutamine, and began to consume a small amount of glucose after 60 h cultivation. Under the 5-FU treatment, glutamine was still the main energy source in 2D monolayer culture, while cells consumed glutamine and glucose at the same time in 3D MTSs. In 3D MTSs, cellular energy metabolism shifted from TCA cycle to glycolysis, and as a result, more glucose was consumed. In addition, with 5-FU treatment reabsorption phenomenon was found in the 3D MTSs, while HeLa cells continued to secrete ammonia under 2D monolayer culture conditions (Fig. 3g). It has been reported that the reabsorption and reuse of ammonia was beneficial to the growth of tumor cells (Spinelli et al. 2017). The secretion rate of lactate in 3D MTSs was slightly higher than that in 2D culture under both control and 5-FU treatment conditions (Fig. 3h, Additional file 1: Fig. S4).

### Identification of transcriptional alterations for 5-FU resistance

**Fig. 4:**
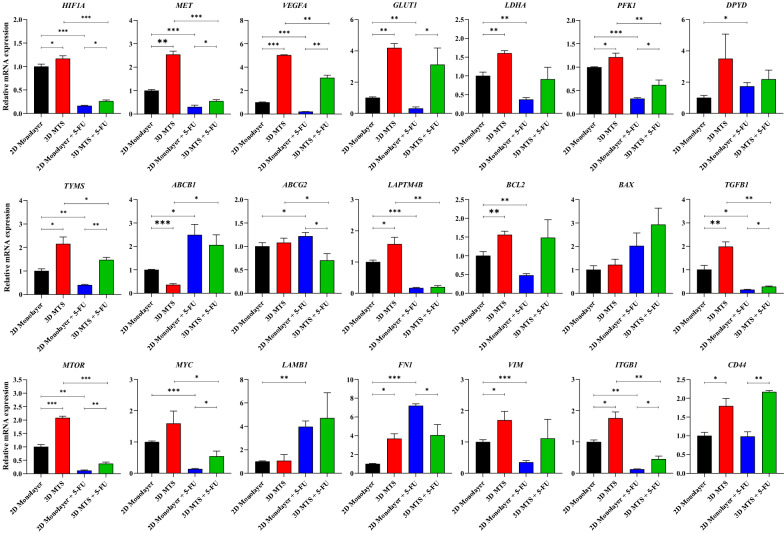
Transcriptional analysis of putative drug resistance genes associated with *HIF1A*-induced signal pathways, glucose metabolism, nucleotide metabolism, multidrug resistance-related gene, ECM secretion, tumor stem cell and signaling pathways related to cell growth and proliferation

The biological characteristics of 3D MTSs were more representative than that of 2D monolayer culture, such as hypoxic regions and pH gradients caused by mass transfer limitations, enhanced ECM secretion, drug permeation barriers caused by closely cells contact, increased ECM deposition and the improvement of tumor cell stemness (Dittmer and Leyh 2015). Therefore, we measured the transcriptional levels of drug resistance-related genes in HeLa cells cultured under both 2D cell cultures and 3D MTSs (Fig. 4).

In the 3D MTS model, there is often an oxygen diffusion limit of 150–200 μm (Oldham et al. 2015). Hence, exceeding this radius would likely form an area of hypoxia in the MTSs, which would lead to genetic and metabolic reprogramming regulated by hypoxic induction factor (*HIF1A*), enabling tumor cells to acquire a more aggressive phenotype (Denko and Nicholas 2008; Zhang et al. 2020). Compared with 2D monolayer cells, after the 5-FU treatment, the transcript level of *HIF1A* in the 3D MTSs were significantly increased by 1.60 times (Fig. 4). It has been reported that tumor hypoxia microenvironment would cause abnormal activation of the oncogene *MET* (Stella et al. 2010), and consistent with this, the transcription level of *MET* was 2.54 and 1.8 times higher up-regulated in the 3D MTSs than in the 2D cultures under the control and 5-FU treatment conditions, respectively, which would promote angiogenesis and maintain tumor aggressiveness. *HIF1A* could also induce the expression of vascular endothelial growth factor (*VEGFA*), which is central to the growth and metastasis of tumors, thereby promoting the malignant progression of tumors (Mohamed et al. 2020). As evidenced, we observed that the transcription level of *VEGFA* was about 5.04 times and 14.87 times higher up-regulated in more 5-FU resistant 3D MTSs than 2D monolayer cells under the control condition and the 5-FU treatment conditions, respectively.

Compared with normal cells, tumor cells display up-regulated glycolysis for the provision of intermediates for rapid proliferation, which is mainly manifested by enhanced glucose uptake and lactate excretion (Yong et al. 2019). Similarly, we observed an increase in glucose utilization and lactate excretion in 3D MTSs, especially following the 5-FU treatment, compared with 2D monolayer culture (Fig. 3e, h). Therefore, the transcript level of the genes encoding glucose transporter (*GLUT*), lactate dehydrogenase (*LDH*) and phosphofructokinase (*PFK1*) were measured. As compared with the 2D monolayer cultures, the transcript levels of *GLUT1*, *LDHA*, *PFK1* were 4.20, 1.60 and 1.21 times higher up-regulated in the 3D MTSs under the control conditions (Fig. 4). As expected, the transcript levels of these genes were more pronouncedly increased after the 5-FU treatment, showing that 9.72, 2.45 and 1.88 times higher up-regulated in the 3D MTSs than in the 2D cultures. This result indicated that 3D MTSs featured enhanced aerobic glycolysis, i.e., the well-known Warburg effect.

The rapid proliferation of tumor cells required a large number of nucleic acids. The only source of thymine in cells is the de novo synthesis pathway, and the high expression of *TYMS* is often associated with poor prognosis of tumors (Donner et al. 2019). 5-FU blocks DNA synthesis to induce cell death by inhibiting TYMS, while dihydropyrimidine dehydrogenase (DPYD) could decompose and deactivate 5-FU before it was converted into active metabolites (Negarandeh et al. 2020). Compared with the 2D cultures, the transcription levels of *TYMS* were 2.15 and 3.48 times higher up-regulated, meanwhile the transcript levels of *DPYD* were 3.66 and 1.26 times higher up-regulated in 3D MTSs before and after the 5-FU treatment conditions, respectively (Fig. 4). Therefore, the up-regulation of the expression of *TYMS* and *DPYD* were also the reasons for the enhanced resistance of MTSs to 5-FU.

Increasing evidence has ever shown that conventional cancer chemotherapy is seriously limited by the multidrug resistance (MDR) commonly exhibited by tumor cells (Perez-Tomas 2006). In drug-resistant tumor cells, the main mechanism was the drug accumulation and efflux in which the ATP binding cassette (ABC) transporters played an important role (Orlando and Liao 2020; Ye et al. 2016). However, we did not observe the up-regulation of *ABCB1* and *ABCG2* transcription levels in 3D MTSs as expected, but there may be differences in protein or metabolite levels. Lysosome-associated transmembrane protein 4B (*LAPTM4B*), a multidrug resistance gene, could stimulate drug resistance and promote cell growth and proliferation by regulating drug efflux mechanism and activating PI3K/Akt signal transduction (Gu et al. 2020). Consistent with this, compared with the 2D monolayer cultures, the expression levels of *LAPTM4B* were 1.57 and 1.32 times higher up-regulated in 3D MTSs before and after the 5-FU treatment (Fig. 4).

Apoptosis defects were also one of the reasons for drug resistance, which were usually regulated by the BCL-2 protein family (Warren et al. 2019). Compared with 2D monolayer cells, the transcription level of the *BCL2* encoding anti-apoptotic protein in 3D MTSs was 1.55 times and 3.09 times higher up-regulated before and after the 5-FU treatment, respectively, which contributed to the progression of tumor drug resistance (Fig. 4). However, the transcription level of the *BAX* encoding apoptotic protein was also up-regulated in 3D MTSs, which might be related to the decrease of cell proliferation activity.

Then, the expression of cytokines related to tumor cell proliferation and progression was tested. Transforming growth factor (*TGFB1*) was generally up-regulated in tumor cells, and could induce epithelial–mesenchymal transition and promote tumor cell growth, proliferation and invasion (Fuxe and Karlsson 2012). The mTOR pathway was a classical signal transduction pathway regulating cell growth and metabolism, and was dysregulated in many cancers (Caron et al. 2010). It has been reported that the glycolytic pathway was affected by the mTOR pathway through two key transcription factors, HIF1A and MYC (Renner et al. 2017). As compared with the 2D monolayer cultures, the transcript levels of *TGFB1*, *MTOR*, *MYC* were 1.92, 2.08 and 1.59 times higher up-regulated in the 3D MTSs, respectively (Fig. 4). As expected, the transcript levels of these genes were more pronouncedly increased after the 5-FU treatment, showing that 1.82, 3.21 and 3.51 times higher up-regulated in 3D MTSs than in the 2D monolayer cultures, respectively (Fig. 4).

ECM, such as laminin, fibronectin, vimentin, mediated interactions between cells and participated in signal transduction in processes such as cell adhesion, migration, invasion, proliferation and EMT to promote the development of drug resistance, referred to as cell adhesion mediated drug resistance (CAM-DR) (Baltes et al. 2020; Valkenburg et al. 2018). Although the transcript level of laminin β1 (*LAMB1*) did not change significantly, compared with the 2D cultures, we found 3.68 times higher up-regulation in the expression of fibronectin (*FN1*) in 3D MTSs under the control condition. Meanwhile, the transcript levels of vimentin (*VIM*) were 1.70 and 3.18 times higher up-regulated in 3D MTSs under the control and 5-FU treatment conditions, respectively (Fig. 4). It was found that ECM participated in CAM-DR by stimulating integrin mediated PI3K activation to protect tumor cells from damages caused by radiotherapy and chemotherapy (Mohanty et al. 2020; Hodkinson et al. 2007). Compared with the 2D monolayer cultures, the expression level of integrin β1 (*ITGB1*) was 1.76 and 3.59 times higher up-regulated in 3D MTSs before and after the 5-FU treatment, respectively (Fig. 4). *ITGB1* (also known as *CD29*) and *CD44* are reported as tumor stem cell markers, and the presence of tumor stem cells could affect the drug treatment and subsequent tumor recurrence (Tomasetti et al. 2017). It has been shown that *CD44* can mediate the stemness of tumor cells and participate in metastasis by binding to hyaluronic acid (Gomez et al. 2020). Compared with 2D monolayer cultures, the expression level of and *CD44* was 1.79 and 2.21 times higher up-regulated in 3D MTSs than in 2D monolayer culture under the control and 5-FU treatment conditions, respectively (Fig. 4).

### Proteome analysis

Tumor cells synthesize a variety of proteins that interact with each other to perform cellular functions through transcriptional, translational and post-translational modifications (Manzoni et al. 2018). Genetic variation and changes of the tumor microenvironment would affect the expression, structure and interaction of proteins, leading to changes in the activities of cells. The differences in protein expression levels between 2D monolayer cultures and 3D MTSs cultures before and after the 5-FU treatment were determined through Tandem Mass Tag (TMT) technology. The PCA result showed that there were significant differences among all sample groups and good repeatability within the sample group (Additional file 1: Fig. S5A), which proved that the obtained experimental data could support the following data analysis. A total of 5262 proteins were determined, and the number of differential proteins is shown in Table 1. We found that the effect of 16 μM 5-FU on the expression profile of protein in 3D MTSs was less pronounced than that in 3D monolayer cells. Under the 5-FU treatment, there were 133 proteins significantly different in 2D monolayer cells compared with the control condition, while only 27 differential proteins were significantly changed in the 3D MTSs, which indicated that 5-FU-induced response in 3D MTS was attenuated. Our results showed that after the 5-FU treatment, there were significant differences in cellular processes related to cell growth and death (e.g., cell senescence, mitophagy, p53 signaling pathway) under 2D monolayer culture (Additional file 1: Fig. S5C, D). However, the differential proteins in the 3D MTSs were not enriched in specific metabolic pathways. While up-regulating the negative cell cycle regulatory protein RB1 to block the cell cycle, 2D monolayer cells up-regulated cyclin (CCNB1, CCNB2), apoptosis inhibitor BIRC5 to maintain growth activity with the 5-FU treatment (Additional file 1: Fig. S5B). In the 3D MTSs, we also observed the up-regulation of BIRC5 with the 5-FU treatment. Meanwhile, the 5-FU treatment reduced the absorption and transport of glucose, folate and amino acids in 2D monolayer cultures, and down-regulated the members of solute carrier family members, including SLC2A1, SLC19A1, SLC38A1 and SLC38A2, which were not significant in the 3D MTSs (Additional file 1: Fig. S5B).

**Table 1:** The number of differential proteins between 2D monolayer culture and 3D MTS before and after the 5-FU treatment

Control group	Experimental group	Up	Down	Sum
2D monolayer	3D MTS	206	161	367
2D monolayer + 5-FU	3D MTS + 5-FU	185	103	288
2D monolayer	2D monolayer + 5-FU	51	82	133
3D MTS	3D MTS + 5-FU	8	19	27

**Fig. 5:**
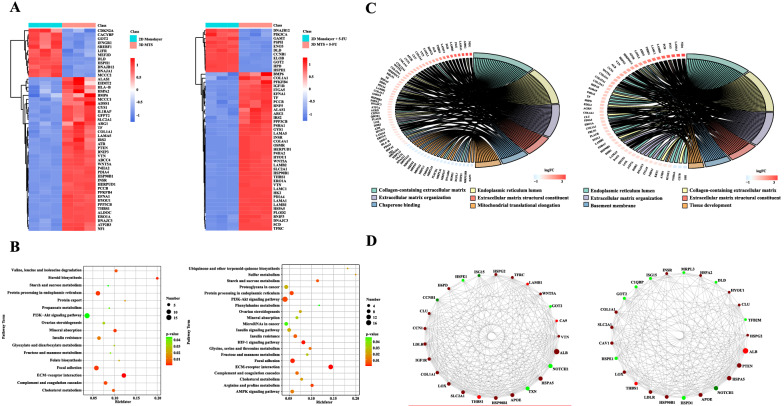
Proteomic differences between cells in 2D monolayer and 3D MTS. **a** Heatmap of differential proteins between 2D monolayer and 3D MTS. 50 differential proteins in the top KEGG pathways (sorted according to the -log10 *p*-value) were selected to draw the heat map. **b** The KEGG pathways involving proteome difference between 2D monolayer and 3D MTS (*p*-value < 0.05, top 20 sorted according to the -log10 *p*-value). **c** Chord diagram of GO analysis. The top 6 GO terms with the number of differential proteins between 3 and 50 were selected (sorted according to the -log10 *p*-value in descending order). The relationship between the selected GO terms and the corresponding differential proteins were shown in the chord diagram. **d** Protein–protein interaction network of the top 25 proteins in connectivity degree. The molecular chaperones related to protein folding, assembly, and secretion exerted the most obvious effects, and the heat shock protein family was dominant. Left, before the 5-FU treatment; Right, after the 5-FU treatment

Table 1 shows 367 and 288 differential proteins between 2D monolayer cultures and 3D MTS before and after the 5-FU treatment, respectively. Based on this, we selected 50 differential proteins which exerted great influence on the growth, metabolism and drug resistance of tumor cells, and then their expression levels were hierarchically clustered between 2D monolayer cultures and 3D MTSs before and after the 5-FU treatment. The differential proteins between 2D monolayer cultures and 3D MTSs displayed similar profiles (Fig. 5a). In order to further analyze metabolic differences, KEGG pathway enrichment analysis was performed with all differential proteins between 2D monolayer cultures and 3D MTSs (Fig. 5b, Additional file 1: Fig. S5E). We observed that ECM proteins were extremely significantly up-regulated in 3D MTSs, including laminin (e.g., LAMA5), collagen (e.g., COLO1A1) and vitronectin (VTN). Processes related to protein synthesis, processing and transportation, which were mainly involved in heat shock protein family, also exhibited significant differences between 2D monolayer cultures and 3D MTSs. In addition, there were significant differences in the mutual conversion of amino acids, in which mitochondrial glutamate oxaloacetic acid transaminase (GOT2) was significantly down-regulated in 3D MTSs. GOT2 plays an important role in amino acid metabolism and TCA cycle (Yang et al. 2015). Consistent with the transcription level (Fig. 4), we found more multidrug resistance-related proteins (ABCC4) in 3D MTSs. Similarly, under the 5-FU treatment, we also found that integrin α5 (ITGA5) was up-regulated in 3D MTSs (Fig. 5a), which was consistent with the up-regulated transcription level of *ITGB1* (Fig. 4). Notably, glucose transport-related proteins, such as SLC2A1, were significantly up-regulated, which further proved the possibility of up-regulation of glycolytic flux of 3D MTSs. In KEGG pathway enrichment analysis, we found that signaling pathways such as PI3K/Akt and HIF-1 were significantly altered (Fig. 5b, Additional file 1: Fig. S5E). In this study, HYOU1 in 3D MTSs was also significantly up-regulated regardless of the 5-FU treatment (Fig. 5a). Hypoxia up-regulated 1 (HYOU1), a member heat shock protein 70 family, maintains endoplasmic reticulum (ER) homeostasis under hypoxia conditions while promotes growth, metastasis and invasion of tumor cells by activating the PI3K/Akt signaling pathway (Li et al. 2019).

Through GO analysis, the gene functions of the differential proteins were analyzed (Fig. 5c, Additional file 1: Tables S3 and S4). Under both control and 5-FU treatment conditions, the differential proteins between 2D monolayer cultures and 3D MTSs were similar in enrichment levels. The molecular functions of 3D MTSs were mainly performed by up-regulating proteins related to calcium ion binding, ECM constituent, integrin binding, and down-regulating proteins related to ATP binding, RNA binding, ubiquitin protein ligase binding. While for the biological process, 3D MTSs mainly up-regulated processes related to ECM organization, cellular protein metabolic process tissue development, blood vessel development and protein folding in endoplasmic reticulum, and down-regulated processes related to mitochondrial function, including mitochondrial translation, mitochondrial transcription, mitochondrial electron transport, etc. In addition, with the 5-FU treatment, MTSs also up-regulated proteins related to cell adhesion and down-regulated proteins related to cell division and cell redox homeostasis. On the other hand, for cellular components, the proteins up-regulated in MTSs were mainly distributed in ER or extracellular regions such as exosomes, ECM, basement membrane, while the down-regulated proteins were mainly distributed in the mitochondria. This is reasonable that the increase of the production and deposition of ECM enhanced the physical barrier of drug penetration, as well as involved in the regulation of the EMT process and the expression of tumor cell stem genes, thereby promoting the development of tumor resistance (Dominijanni et al. 2020; Joyce et al. 2018). Mitochondrion, an important signal transduction hub, can regulate cell apoptosis and participate in cell communication and tumor formation through ROS, nitric oxide, and calcium ions and proteins involved in the apoptotic cascade (Frezza 2014). It has been reported that the down-regulation of mitochondria-related genes promoted the up-regulation of EMT pathway and promoted the invasion and metastasis of tumor cells (Gaude 2018). Therefore, the up-regulation of ECM construction and the down-regulation of mitochondrial function genes might lead to the enhanced resistance of 3D MTSs to 5-FU. The interactions between differential proteins were further analyzed using the STRING database for the protein–protein interaction network (Fig. 5d), and we found that the molecular chaperones related to protein folding, assembly, and secretion exerted the most obvious effects, and the heat shock protein family was dominant (such as, HSPA5, HSP90B1, HSPG2). The increased expression of molecular chaperones was conducive to maintaining the growth of tumor cells in unfavorable environments, and the overexpression of HSP90 is related to the poor prognosis response of tumors and the increase of drug resistance (Jarosz 2016).

### Metabolome analysis

The metabolome could directly reflect the physiological characteristics of cells, and the minor changes in gene transcription and protein expression would cause significant differences in the metabolome (Klein and Heinzle 2012). Tumor cells often alter signal transduction pathways and rewire metabolism, thereby promoting cell proliferation and weakening the effect of anticancer drugs (Locasale 2012). In this study, we analyzed 330 metabolites, including carbohydrates, amino acids, lipids, nucleotides, organic phosphates, sterols, coenzymes of tumor cells cultured under both 2D monolayer cultures and 3D MTSs before and after the 16 μM 5-FU treatment. The PCA result showed the significant differences among groups and good repeatability in groups, which proved that the following data analysis could be supported (Additional file 1: Fig. S6A). Table 2 shows 107 and 76 differential metabolites under 2D monolayer cultures and 3D MTS before and after the 5-FU treatment, respectively. With the 5-FU treatment, the metabolites involved in the ABC transport system of cells were significantly different between 2D monolayer cells and 3D MTSs (Additional file 1: Fig. S6B–D). The effects of 5-FU on sugar metabolism and amino acid metabolism in 3D MTSs were less pronounced than in monolayer cell culture. Under the 5-FU treatment, the intermediates of TCA cycle (including citrate, malate and fumarate) and of alanine, aspartate and glutamate metabolism were all decreased in 2D monolayer cells, while there were no significant differences in 3D MTSs, except for the increase of asparagine. Similarly, the effect of 5-FU on the nucleotide metabolism of MTSs was also relatively weakened. 5-FU disrupts the de novo nucleotide synthesis pathway by targeting TYMS (Longley et al. 2003). Under the 5-FU treatment, the nucleotide synthesis pathway of 2D monolayer cells was inhibited with the increase of 5-phospho-ribulose, while purine nucleotide synthesis was blocked with the increase of purine synthesis precursors inosine and hypoxanthine and the decrease of inosine monophosphate (IMP) and adenosine monophosphate (AMP). It was also found that the precursor orotate to uracil synthesis was decreased in 2D monolayer cells with the 5-FU treatment. Under the 5-FU treatment, we find the decrease of inosine, xanthine and orotate, and the increase of uridine. After 5-FU treatment, the pentose phosphate pathway (PPP) changed significantly. Under 2D culture conditions, gluconolactone and gluconic acid were significantly increased, while 6-phosphogluconic acid was significantly decreased, possibly due to the increased production of ribulose 5-phosphate (Additional file 1: Fig. S6B). This process is accompanied by the production of NADPH (Cairns et al. 2011), which contributes to the elimination of ROS induced by 5-FU (Fig. 3d). In 3D MTSs, we also observed an increase in ribulose 5-phosphate, which resulted in a significant decrease in gluconolactone and gluconic acid (Additional file 1: Fig. S6B).

**Table 2:** The number of differential metabolites between 2D monolayer culture and 3D MTS before and after the 5-FU treatment

Control group	Experimental group	Up	Down	Sum
2D monolayer	3D MTS	44	55	99
2D monolayer + 5-FU	3D MTS + 5-FU	56	35	91
2D monolayer	2D monolayer + 5-FU	27	80	107
3D MTS	3D MTS + 5-FU	41	35	76

**Fig. 6:**
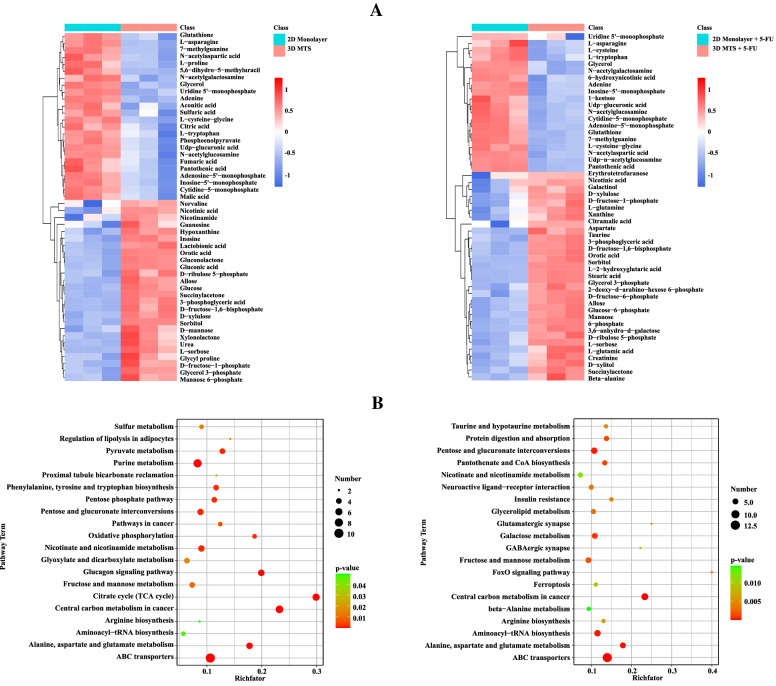
Metabolomic differences between cells in 2D monolayer and 3D MTS. **a** Heatmap of differential metabolites between 2D monolayer and 3D MTS. 50 differential metabolites in the top KEGG pathways (sorted according to the -log10 *p*-value) were selected. **b** The KEGG pathways involving metabolome difference between 2D monolayer and 3D MTS (*p*-value < 0.05, top 20 sorted according to the -log10 *p*-value). Left, before the 5-FU treatment; Right, after the 5-FU treatment

In the control conditions, there were 99 different metabolites between 2D and MTSs while 91 different metabolites were detected under the condition of the 5-FU treatment. We selected 50 differential metabolites which exerted greater impacts on tumor cell growth, metabolism, and drug resistance, and then their expression levels were hierarchically clustered between 2D monolayer cultures and 3D MTSs under both control and 5-FU conditions (Fig. 6a). KEGG enrichment was performed to examine the differences in metabolic pathways of tumor cells under different culture conditions (Fig. 6b, Additional file 1: Fig. S6E). The ABC transport system played an important role in metabolite transport and was associated with the multidrug resistance (Nunes et al. 2019). We found that the metabolites involved in the ABC transport system were significantly different between 2D monolayer cultures and 3D MTSs regardless of the 5-FU treatment, which may indicate the differences in drug transport capacity. The carbon metabolism pathways performed significant differences in 2D monolayer cultures and 3D MTSs. Under control conditions, compared with monolayer cells, the TCA cycle intermediates including citric acid, cis-aconitic acid, succinic acid, fumaric acid, malic acid were decreased in 3D MTSs, which was consistent with the significant increase in cellular non-mitochondrial respiration and the down-regulated protein expression related to mitochondrial activity (Fig. 3c, Additional file 1:Tables S3 and S4). However, we did not observe significant differences in the intermediate metabolites of the glycolysis pathway between 2D monolayer cultures and 3D MTSs. This indicated that the flux control may be governed by other regulation mechanisms, e.g., phosphorylation-mediated reprogramming of glycolytic activity (Ruprecht et al. 2017). We found that there were no significant differences in glutamine and glutamate between 2D monolayer cultures and 3D MTSs, while with 5-FU treatment, glutamine and glutamate significantly increased in 3D MTSs (Fig. 6a). Glutamine participated in the TCA cycle by converting to glutamate and then further to α-ketoglutarate, which maintained the TCA cycle in 3D MTSs under the 5-FU treatment, resulting in no significant difference in the TCA cycle between 2D culture and 3D MTSs after 5-FU treatment. Meanwhile, asparagine significantly decreased under both conditions in the 3D MTSs, which led to the decrease of aspartate involved in the TCA cycle. Furthermore, compared with 2D culture, gluconolactone, gluconic acid and ribulose 5-phosphate involved in the PPP were significantly increased in 3D MTSs under the control condition; under the 5-FU treatment, only the significant increase of ribulose 5-phosphate was found in 3D MTSs.

## Discussion

### Generation of reproducible 3D MTSs using an optimized liquid overlay method

Up to now, researchers have established a variety of methods for the preparation of MTSs (scaffold-based or scaffold-free), among which the liquid overlay method based on non-adhesive surfaces has been the most popular technique for the MTS production due to its generality and low cost (Costa et al. 2018). Different culture methods could be combined with each other, and external forces, such as centrifugation, magnetic fields, electric fields, mechanical vibrations, acoustic waves, are conducive to cell aggregation (Anggayasti et al. 2020). Based on the liquid overlay method, in this study we optimized the MTS culture process and established a rapid, efficient and reproducible MTS generation method (Additional file 1: Table S2). The tightness and compactness of MTSs reflect the strength of the interaction between cells and the density of cells accumulation, which directly affects cell function, drug penetration and drug response (Koudan et al. 2020). The introduction of the whole well-plate centrifugation and Matrigel™ additives significantly improved the roundness and particle size uniformity of MTSs, facilitating the formation of tighter and more regular 3D structures. A single MTS was formed in each well of the plates, which allows to conveniently monitor the growth dynamics of MTSs, improves the reproducibility, facilitates multi-omics analysis and high-throughput screening of antitumor drugs. Meanwhile, an obvious observation is that the number of cells at plateau in the form of 3D MTS is dependent on the initial cell concentration. In the process of MTS culture, without changing the medium, cells continue to consume nutrients and secrete metabolites such as lactic acid and ammonium. A relatively longer incubation time and lower cellular growth rate were observed when the initial inoculation density is lower (Fig. 2d). The discrepancy regarding the number of cells at plateau is mainly ascribed to the availability of nutrient uptake and metabolic waste excretion to the surface cell in the 3D MTS where a large initial cell concentration allows more surface cells to achieve this goal. Further, in this study, the initial diameters of 3D MTSs with 1000 and 2000 cells/well exceeded 300 µm, which further set limits for mass transfer in terms of nutrient uptake and excretion of metabolic wastes (Fig. 2b). Meanwhile, the little evaporation of medium may aggravate the formation of unfavorable environment for cell growth. As a result, a growth advantage in the initial stage most probably leads to a larger number of viable cells at plateau. The diameter of MTSs could be controlled at about 500 μm, above which they would stratify to form a necrotic core (Oldham et al. 2015). The reduced proliferative activity (Fig. 2f, Additional file 1: Figure. S3) and blocked cell cycle in 3D MTSs (Fig. 3b) may contribute to the development of resistance to 5-FU (Maier et al. 2018). This method was suitable for the 3D culture of a variety of tumors, and could also be used for co-culturing with stromal cells and immune cells to construct more complex 3D tumor models. HeLa cells cultured in 3D MTSs showed enhanced 5-FU resistance, with a drug resistance index of about 5.72. Therefore, this study provided a scalable method for 3D MTS generation, which benefits the study of tumor drug resistance mechanism and the application of high-throughput drug screening.

### Metabolic shift toward glycolysis in 5-FU-resistant HeLa cells

It has been reported that the increase of glycolytic pathway in melanoma cells promotes the growth and invasion of tumor, mediates stronger drug resistance, and impaired T cell killing of tumor cells (Cascone et al. 2018). Mitochondrial dysfunction and hypoxia are two important factors that induce the Warburg effect (Xu et al. 2005). In the 3D MTSs, the utilization of glucose was increased compared with 2D monolayer cultures (Fig. 3e) and the expression of GLUT1, SLC2A1 and LDHA were also up-regulated (Figs. 4, 5a), which indicated the enhanced glycolytic flux. The non-mitochondrial respiration of MTSs was significantly improved (Fig. 3c). Through comparative proteome and metabolome analysis (Figs. 5a-d, 6a, b), we found that in 3D MTSs the biological processes related to the mitochondrial function were significantly down-regulated, and the intermediates involved in TCA cycle were decreased in 3D MTSs, but there were no significant differences in the enzymes and intermediate metabolites of the glycolytic pathway. Ruprecht et al. have shown that post-translational modifications of glycolysis, especially phosphorylation, lead to glycolysis addiction and mediate drug resistance (Ruprecht et al. 2017). The existence of hypoxic regions of 3D MTSs restricted mitochondrial respiration, leading to incomplete oxidation of nutrients, and forcing tumor cells to up-regulate the glycolysis as the primary pathway for energy supply. Previous study has demonstrated that mitochondrial function as oxygen sensors and releasing ROS can stabilize the signal hypoxia induced factors such as HIF-1α and HIF-2α (Guzy et al. 2005). Consistent with this, the ROS levels are significantly higher in the 3D MTS than in the 2D monolayer cultures (Fig. 3d). Also, we observed that 5-FU significantly induced the formation of the ROS under both 2D monolayer cultures and 3D MTSs (Fig. 3d). The continuous accumulation of ROS would further cause damage to mitochondrial DNA and electron transport chain, aggravating mitochondrial dysfunction and dependence on the glycolysis (Pelicano et al. 2004). Tumor cells relying on glycolysis produce large amounts of lactate, which was indeed observed especially after the 5-FU treatment (Fig. 3H). It was shown that the secretion of lactate could protect cells from damage under hypoxia conditions, promote angiogenesis and cell growth, and also contribute to activate MET-dependent signal transduction pathways in tumor cells to promote drug resistance (Apicella et al. 2018; Lee et al. 2015). Reducing the ratio of extracellular acidification rate and oxygen consumption rate of tumor cells would contribute to reducing the cell invasion ability and the development of drug resistance (Yizhak et al. 2014). Therefore, the metabolic shift of 3D MTSs to glycolysis may contribute to the increased resistance to 5-FU.

### Maintenance of endoplasmic reticulum homeostasis in 5-FU-resistant HeLa cells

The changes of chaperone-mediated folding play an important role in tumorigenesis and the evolution of drug resistance mechanisms (Jarosz 2016). Based on proteomic profiling, we found that the most significant difference in protein synthesis, processing and transport between 2D monolayer cultures and 3D MTSs was mainly enriched in the ER, which was dominated by the heat shock protein family, including HYOU1, HSPA5, HSP90B1, HSPG2 (Fig. 5A–D). Compared with 2D monolayer cultures, changes in the tumor microenvironment and metabolic rearrangement of tumor cells in 3D MTSs would affect protein processing (Mischiati et al. 2015). It was shown that hypoxia areas in tumor, nutritional deficiency, pH gradient, and disorder of calcium ion homeostasis could promote unfolded protein response (UPR) to activate the ER stress response (Ron and Walter 2007). UPR signaling can contribute to maintaining proper protein folding function under the ER stress, but the mechanism of apoptosis would be triggered when the cells were exposed to ER stress for a long time and the ER function was impaired (Hoyer-Hansen and Jaattela 2007). We observed an up-regulation of protein folding, which contributes to the maintenance of ER homeostasis (Additional file 1: Tables S3 and S4). Pavan et al. showed that disrupting ER homeostasis by increasing the concentration of calcium ion could induce the death of tumor cells (Pavan Grandhi et al. 2017). Thus, the maintenance of ER homeostasis in 3D MTSs may be one of the major reasons for the increased drug resistance to 5-FU.

### Up-regulation of extracellular matrix in 5-FU resistant HeLa carcinoma cells

ECM, a complex network which is composed of a variety of macromolecules surrounding cells, provides cells with spatial structure and physical support, mediates multiple signal pathways, and plays an important role in cancer progression, invasion and metastasis, and drug resistance (Langhans 2018). Integrin could be involved in CAM-DR by binding to ECM (e.g., collagen I) and activate drug efflux of ABC transporters to develop drug resistance (Baltes et al. 2020). It was found that ECM participated in the regulation of cell cycle by stimulating integrin mediated PI3K activation to protect small cell lung cancer from damages caused by radiotherapy and chemotherapy (Hodkinson et al. 2007). Consistent with these findings, we found in this study the expression of ECM including laminin and collagen was increased in the 3D MTSs (Figs. 4, 5a-d), meanwhile the molecular function of integrin binding was also increased in the 3D MTSs (Additional file 1: Tables S3 and S4), including up-regulation of ITGB1 and ITGA5 on the mRNA and protein level, respectively (Fig. 5a). These significance difference between 2D monolayer and 3D MTSs may contribute to the increase of resistance to 5-FU. Therefore, ECM would become a potential target for improving the effect of anticancer therapy.

## Conclusions

**Fig. 7:**
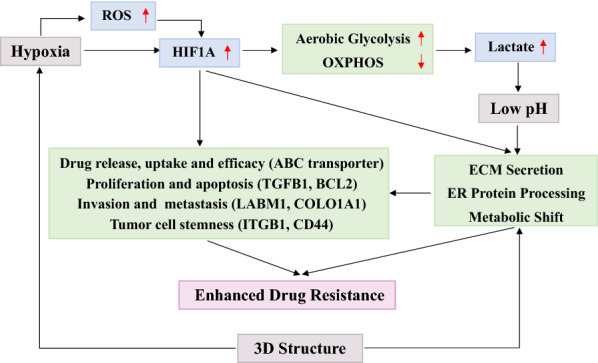
The major mechanisms responsible for the 5-FU resistance in the 3D MTS

In this study, based on the liquid overlay method, we established a rapid and efficient method to produce uniform size and high reproducibility of 3D MTSs. Based on multi-omics data, the phenotypic differences of HeLa cells under different culture conditions were explored based on the established 3D MTS model to investigate the resistance mechanism of 5-FU and discover the key regulatory genes and related reprogrammed metabolic pathways (Fig. 7). We found that in 3D HeLa carcinoma cells the metabolic shift towards glycolysis, the maintenance of ER homeostasis and the up-regulation of ECM may contribute to the formation of increased resistance to 5-FU. In addition, the changes in ABC transporters, HIF-1 signaling pathway and the improvement of tumor cell stemness were also related to the increase of 5-FU resistance of 3D MTSs. Overall, this study demonstrates that resistant 3D Hela MTS can be a promising tool for testing drug delivery and efficacy for cervical cancer treatment, and a multi-omics analysis can reveal metabolic addictions which can be manipulated to restore susceptibility to chemotherapy drugs, since this model is readily compatible with the methodologies and techniques that are already used for the analysis of conventional 2D monolayer cultures.

### Supplementary Information

**Additional file 1.** Details of mass spectrometry-based analytical methods, supplementary figures and tables. **Fig. S1.** The application of 3D MTSs culture method to a variety of tumor cell types and co-culturing models. **Fig. S2.** The morphology and microstructure of multi-component 3D MTSs. **Fig. S3.** The specific growth rates of HeLa carcinoma cells cultured in 2D monolayer and 3D MTS. **Fig. S4.** The specific rates of extracellular (A) glucose, (B) glutamine, (C) ammonium, and (D) lactate. **Fig. S5.** (A) PCA analysis of proteome. (B) Heatmap of differential proteins between the control condition and 5-FU treatment. (C) The KEGG pathways involving proteome difference between the control condition and 5-FU treatment (*p*-value < 0.05, top 20 sorted according to the -log10 *p*-value). (D) KEGG map of differential proteins for 2D monolayer between the control condition and 5-FU treatment. (E) KEGG map of differential proteins between 2D monolayer and 3D MTSs before and after 5-FU treatment. The increase and decrease of proteins are marked with red and green rectangles, respectively. **Fig. S6.** (A) PCA analysis of metabolome. (B) Heatmap of differential metabolites between the control condition and 5-FU treatment conditions. (C) The KEGG pathways involving metabolome difference between the control condition and 5-FU treatment (*p*-value < 0.05, top 20 sorted according to the -log10 *p*-value). (D) KEGG map of differential metabolites for 2D monolayer and 3D MTS between the control condition and 5-FU treatment. (E) KEGG map of differential metabolites between 2D monolayer and 3D MTSs before and after 5-FU treatment. The increase and decrease of metabolites are marked with red and blue circles, respectively. **Table S1.** Primer sequences in this study. **Table S2.** Screening results of MTSs culture conditions. **Table S3.** GO enrichment results between 2D monolayer culture and 3D MTS under the control condition. **Table S4.** GO enrichment results between 2D monolayer culture and 3D MTS under 5-FU treatment condition.

## Data Availability

All data produced or analyzed for this study are included in the published article and its additional information files.
