# Supplementary material for: Multi-omics analysis defines 5-fluorouracil drug resistance in 3D HeLa carcinoma cell model
Source: Bioresour Bioprocess. 2021 Dec 23;8(1):135. doi: 10.1186/s40643-021-00486-z (PMC10991626; doi:10.1186/s40643-021-00486-z)
Supplement: Supplementary file 1 — Additional file 1. Details of mass spectrometry-based analytical methods, supplementary figures and tables. Fig. S1. The application of 3D MTSs culture method to a variety of tumor cell types and co-culturing models. Fig. S2. The morphology and microstructure of multi-component 3D MTSs. Fig. S3. The specific growth rates of HeLa carcinoma cells cultured in 2D monolayer and 3D MTS. Fig. S4. The specific rates of extracellular (A) glucose, (B) glutamine, (C) ammonium, and (D) lactate. Fig. S5. (A) PCA analysis of proteome. (B) Heatmap of differential proteins between the control condition and 5-FU treatment. (C) The KEGG pathways involving proteome difference between the control condition and 5-FU treatment (p-value < 0.05, top 20 sorted according to the -log10 p-value). (D) KEGG map of differential proteins for 2D monolayer between the control condition and 5-FU treatment. (E) KEGG map of differential proteins between 2D monolayer and 3D MTSs before and after 5-FU treatment. The increase and decrease of proteins are marked with red and green rectangles, respectively. Fig. S6. (A) PCA analysis of metabolome. (B) Heatmap of differential metabolites between the control condition and 5-FU treatment conditions. (C) The KEGG pathways involving metabolome difference between the control condition and 5-FU treatment (p-value < 0.05, top 20 sorted according to the -log10 p-value). (D) KEGG map of differential metabolites for 2D monolayer and 3D MTS between the control condition and 5-FU treatment. (E) KEGG map of differential metabolites between 2D monolayer and 3D MTSs before and after 5-FU treatment. The increase and decrease of metabolites are marked with red and blue circles, respectively. Table S1. Primer sequences in this study. Table S2. Screening results of MTSs culture conditions. Table S3. GO enrichment results between 2D monolayer culture and 3D MTS under the control condition. Table S4. GO enrichment results between 2D monolayer culture and 3D MTS under 5-FU treatm [file 40643_2021_486_MOESM1_ESM.docx]

**Supplementary materials**

**Multi-omics analysis defines 5-fluorouracil drug resistance in 3D HeLa carcinoma cell model**

Lin Wang^a^, Xueting Wang^a^, Tong Wang^a^, Yingping Zhuang^a,b^, Guan Wang^a,b^*

a. State Key Laboratory of Bioreactor Engineering, East China University of Science and Technology (ECUST), Shanghai, People’s Republic of China.

b. Qingdao Innovation Institute of East China University of Science and Technology.

*Correspondence: [guanwang@ecust.edu.cn](mailto:guanwang@ecust.edu.cn)

**Mass spectrometry-based analytical methods**

**Chemicals and Materials**

Hydroxylamine was purchased from Sigma-Aldrich (San Luis, MO, USA). Dithiothreitol (DTT), and iodoacetamide (IAA) were obtained from Sangon (Shanghai, CN). Tetraethylammonium bromide (TEAB) was purchased from Sigma. Absolute ethanol and isopropanol were acquired from GENERAL-REAGENT (Titan, Shanghai, CN). HPLC grade methanol, water, acetonitrile, and n-hexane were purchased from CNW (Duesseldorf, GER). Pyridine, formate, acetone, O-methylhydroxylamine hydrochloride, N, O- Bistrimethylsilane trifluoroacetamide (BSTFA), trimethylchlorosilane (TMCS) was purchased from CNW. The internal standard L-2-chlorophenylalanine was obtained from HC Biotch (Shanghai, CN) and fatty acid methyl ester (FAME) internal standard obtained from Larodan (Solna, SWE).

**Proteome analysis**

**Protein extraction:** Added protein lysate containing 1 mM PMSF (P301906, Aladdin, Shanghai, CN), then lysed for 3 min with 1 s of sonication followed by 1 s rest interval. The supernatant was collected after centrifugation at 12000 g for 10 min twice at 4 ℃. Protein concentration was determined with the bicinchoninic acid (BCA) protein assay kit (23227, Thermo Fisher Scientific, Waltham, MA, USA).

**Enzymatic hydrolysis:** Added 5 mM DTT to 50 µg protein from each sample and incubated at 55 ℃ for 30 min. After cooling to room temperature, treated with 10 mM IAA in the dark for 15 min. Added 6 times the sample volume of acetone solution at -20 ℃ overnight to precipitate the protein, centrifuged at 8000 g at 4℃ for 10 min to discard the supernatant, then volatilized the acetone in the fume hood. Added 100 μL of 200 mM TEAB to dissolve the precipitate, then incubated overnight at 37 ℃ with 1 μL of 1 mg / mL of trypsin. The samples were lyophilized and stored at -80 ℃.

**Peptide labeling:** After TMT reagent (90309, Thermo Fisher Scientific, Waltham, MA, USA) was restored to room temperature, 88 μL of anhydrous acetonitrile was added. Added 50 μL of 100 mM TEAB and 41 μL TMT solution to each sample, and reacted at room temperature for 1 h. To stop the reaction, add 8 μL 5% hydroxylamine at room temperature for 15 min. The samples were lyophilized and stored at -80℃.

**Reversed phase liquid chromatography**

All samples were separated using an Agilent 1100 HPLC system with the flow rate of 300 μL/min. The peptide samples were injected onto an Agilent Zorbax Extend-C18 narrow-bore column (1 × 150 mm, 5 μm) with buffer A (2% acetonitrile in water) and buffer B (90 % acetonitrile in water), which adjusted to pH 10 with ammonia water. The separation conditions were as follows: 0 ~ 8 min, 98% A; 8 ~ 8.01 min, 98% ~ 95% A; 8.01 ~ 48 min, 95% ~ 75% A; 48 ~ 60 min, 75 ~ 60% A; 60 ~ 60.01 min, 60 ~ 10% A; 60.01 ~ 70 min, 10% A; 70 ~ 70.01 min, 10 ~ 98% A; 70.01 ~ 75 min, 98% A. The eluent was collected for 8 ~ 60 minutes, and the samples were repeatedly and successively collected in tubes No. 1-15 every minute. Then samples lyophilized prior for LC-MS/MS analysis.

**LC-MS/MS analysis:** All samples analyzed with a Q Exactive HFX mass spectrometer (Thermo Fisher Scientific, Waltham, MA, USA) coupled to an EASY-nLC 1200 liquid chromatograph system (Thermo Fisher Scientific, Waltham, MA, USA) at a flow rate of 300 μL/min. The peptide samples were loaded onto a 75 μm × 20 cm (RP-C18-AQ, reprosil-pur) with buffer A (0.1% formic acid in water) and buffer B (0.1% formic acid in 80% acetonitrile in water). The separation conditions were as follows: 0 ~ 1 min, 1 ~ 6% B; 1 ~ 46 min, 6 ~ 25% B; 46 ~ 52 min, 25 ~ 37% B; 52 ~ 54 min, 37 ~ 95% B; 54 ~ 60 min, 95% B. The parameters of first-order MS were set as follows: The mass resolution was set as 60000, The automatic gain control value was set as 3e6; The maximum injection time was 20 ms; Full MS scans were acquired with an m/z range of 350–1800. The top 15 peaks were scanned by MS/MS using high-energy collision lysis in a data-dependent positive ion mode with the collision energy of 35. The resolution of MS/MS was 45000, the automatic gain control was set as 2e5, the maximum ion injection time was 100 ms, and the dynamic elimination period was set as 30 s.

**Database search**

The LC-MS data was analyzed by Proteome Discover 2.4, then imported into the Uniprot database (http://www.uniprot.org/) for qualitative and quantitative analysis of proteome. In order to improve the reliability of statistical results, the internal data normalization process was carried out during database search.

**Metabolite analysis**

**Metabolite extraction and pre-treatment**

Added 1 mL of pre-chilled 20% methanol, 40 μL internal standard (0.3 mg/mL L-2-chlorophenylalanine, prepared with methanol), and 200 μL chloroform to the sample. Dispersed the sample by ultrasonic for 20 min with 6 s of sonication followed by 4 s rest interval. Centrifuged at 13000 rpm, 4℃ for 10 min, 600 μL supernatant were transfer to the derivatization vial. The supernatant of all samples was mixed in the equal volume to prepare 600 μL of quality control sample (QC), transferred to a derivatization vial, then freeze-dried. Added 80 μL of 15 mg/mL methoxyamine hydrochloride pyridine solution, reacted at 37 ℃ for 90 min. Added 50 μL of N, O-bistrimethyl Silyl trifluoroacetamide/trimethylchlorosilane (BSTFA/TMCS 1000: 1, v/v), 20 μL n-hexane, 10 μL of FAME as internal standard (methyl octoate/methyl nonanoate/methyl decanoate/methyl dodecanoate/methyl myristate, 0.16mg/mL; methyl eicosanate/methyl behenate/methyl tetracosanoate/methyl hexadecanoate, 0.08mg/mL, Chloroform configuration), derivatized at 70 ℃ for 60 min. After cooling to room temperature, GC-MS metabolomics analysis was performed.

**GC-MS condition:** All samples analyzed with an Agilent (Santa Clara, CA, USA) 7890B-5977A with DB-5MS capillary column (30 m × 0.25mm × 0.25 μm, Agilent, Santa Clara, CA, USA) with high purity helium as carrier gas at a flow rate of 300 μL/min. The temperature of the injector was 260 ℃. Temperature programmed: 60 ℃ for 0.5min, 8 ℃/min temperature programmed to 125 ℃; 5 ℃/min temperature programmed to 210 ℃; 10 ℃/min temperature programmed to 270 ℃; 20 ℃/min temperature programmed to 305 ℃, maintained for 5 minutes. The parameters of MS were set as follows: electron impact ion source, ion source temperature 230 ℃, quadrupole temperature 150 ℃, electron energy 70 eV. Full MS scans were acquired with an m/z range of 50-500.

**Database search**

The GC-MS data was analyzed by MS-DIAL 4.7, and all peak signal intensity (peak areas) were normalized according to the internal standard of RSD (Relative standard deviation) < 0.3. After normalization of data, redundancy removal and peak combination were carried out. Then data were imported into the LUG database (Untargeted database of GC-MS from Lumingbio) and NIST database (https://webbook.nist.gov/chemistry/) for qualitative and quantitative analysis of metabolome.

**Supplementary figures**

**
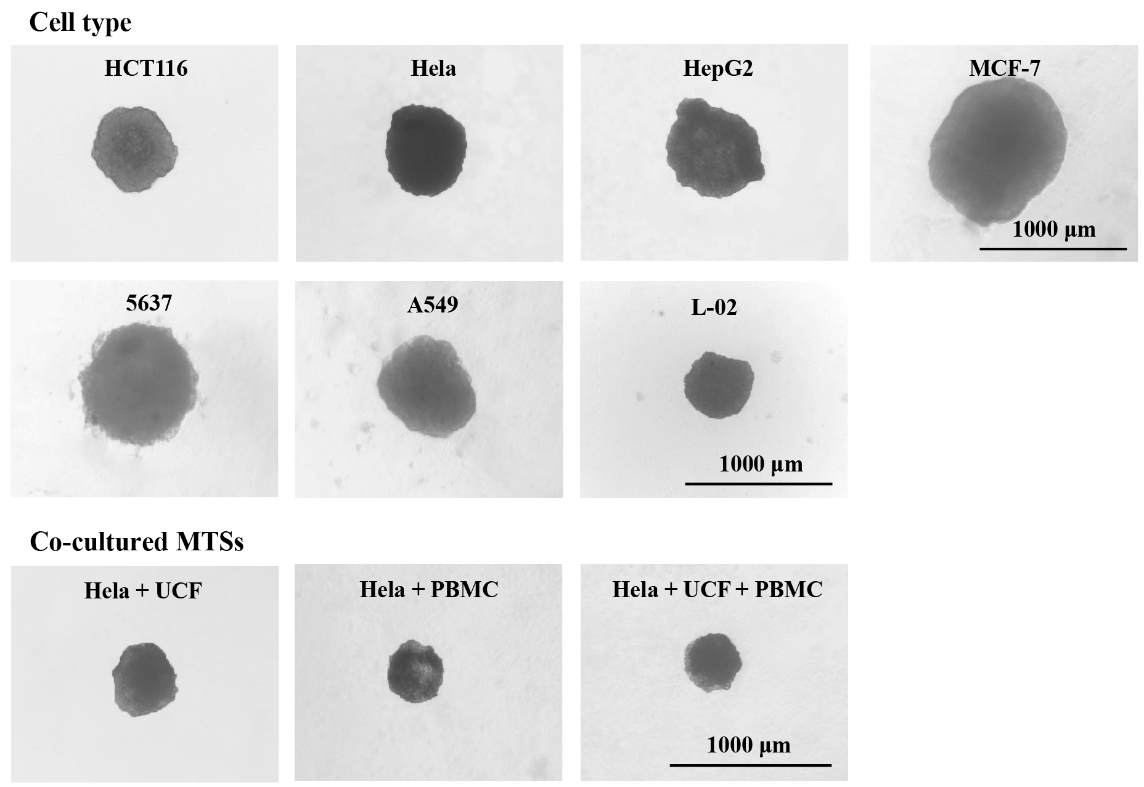
**

**Fig. S1**. The application of 3D MTSs culture method to a variety of tumor cell types and co-culturing models.


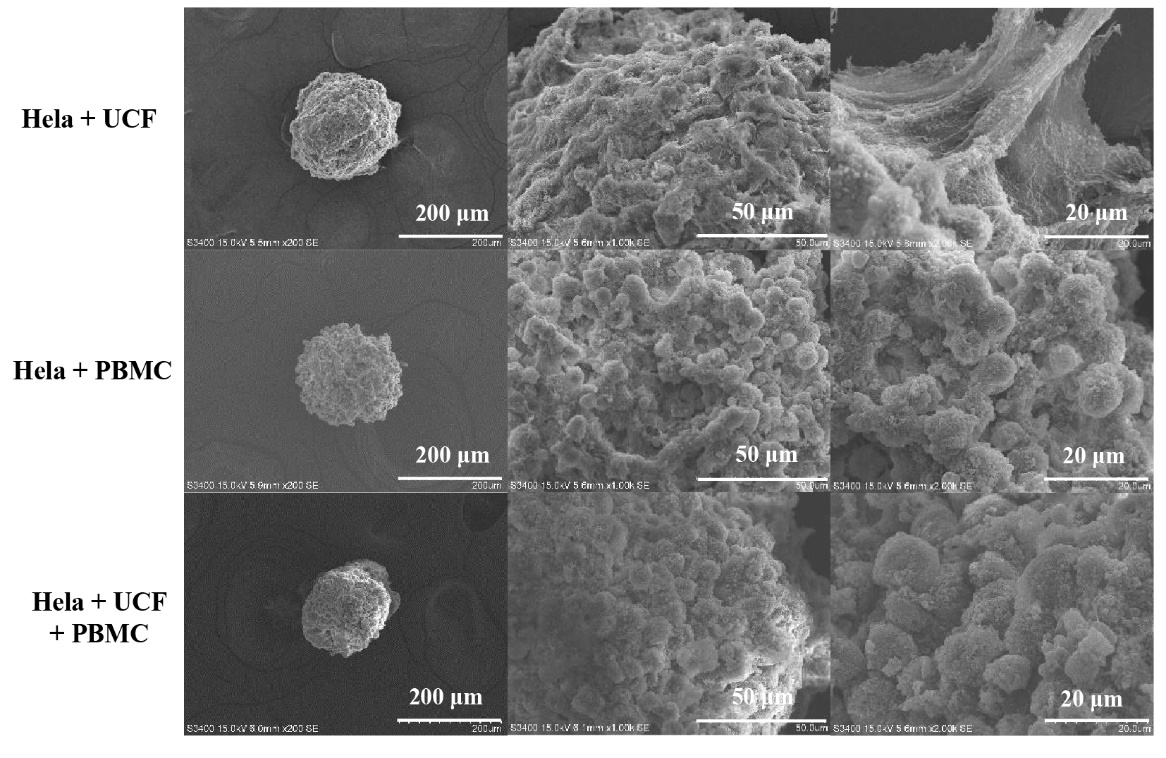


**Fig. S2**. The morphology and microstructure of multi-component 3D MTSs.


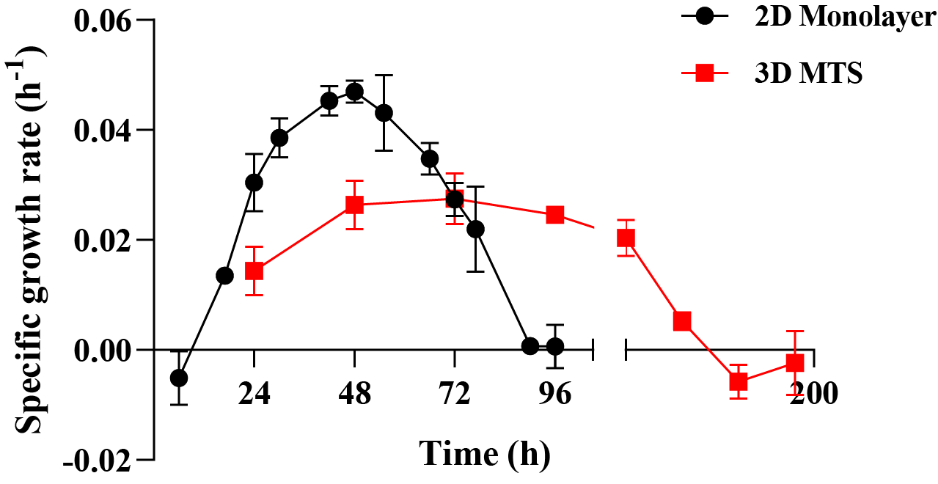


**Fig. S3**. The specific growth rates of HeLa carcinoma cells cultured in 2D monolayer and 3D MTS.


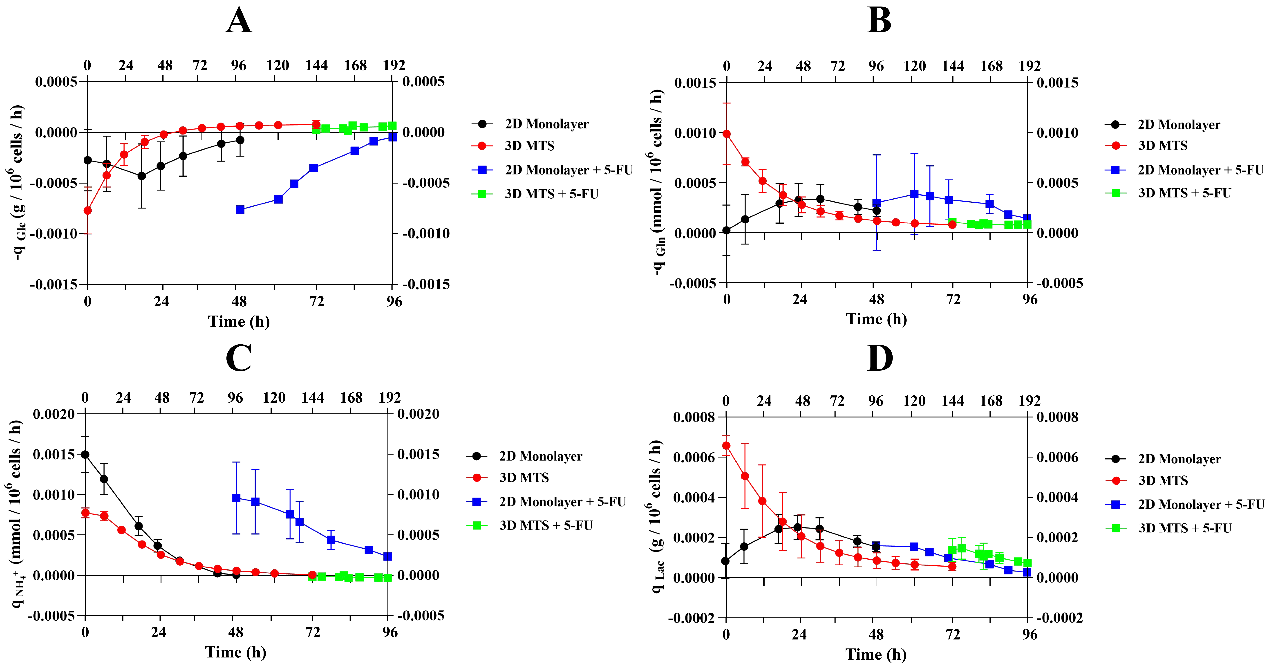


**Fig. S4**. The specific rates of extracellular (A) glucose, (B) glutamine, (C) ammonium, and (D) lactate. The culture time of 2D Monolayer and 2D Monolayer + 5-FU was shown on the bottom X-axis, and the culture time of 3D MTS and 3D MTS + 5-FU were shown on the top X-axis.


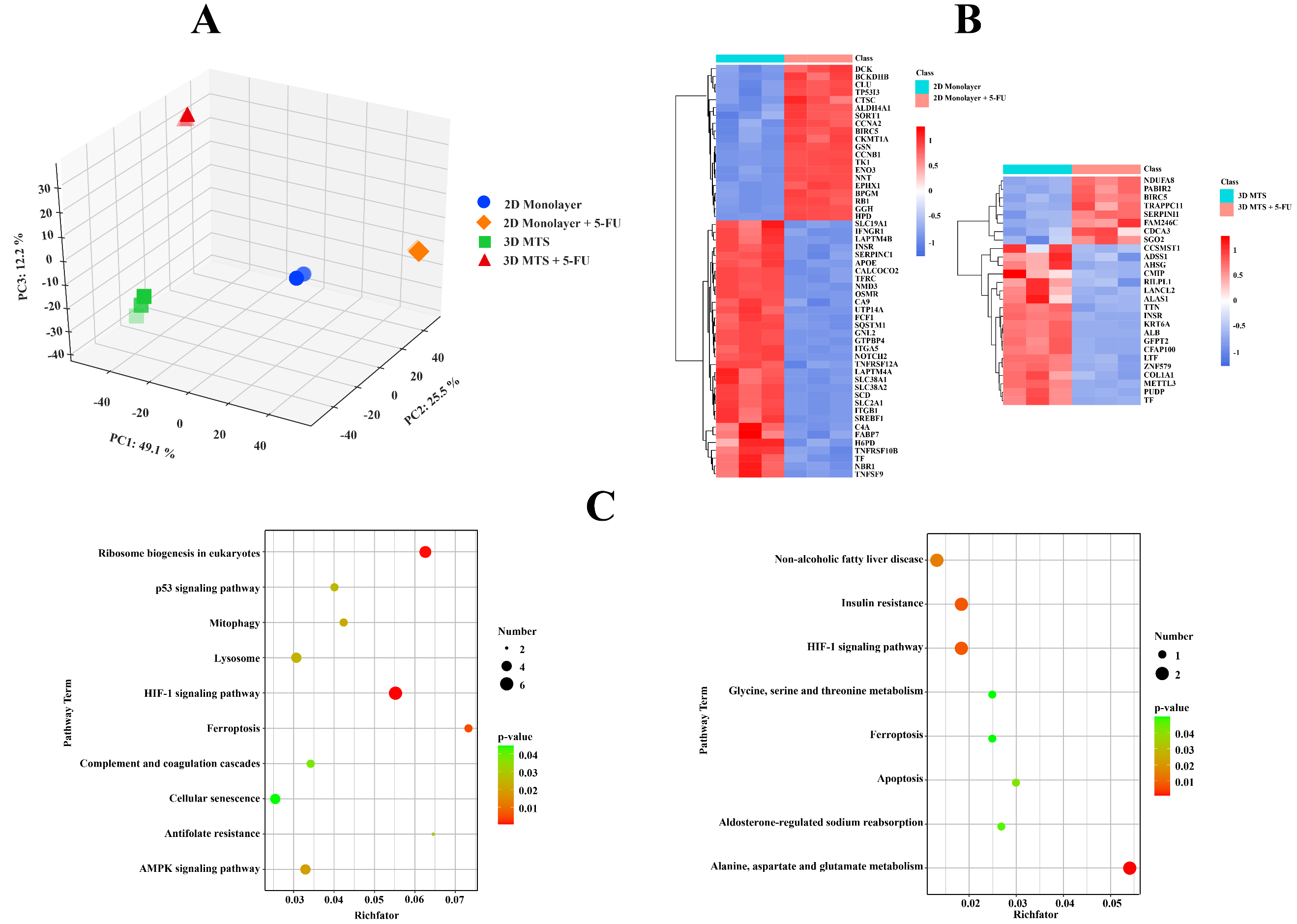


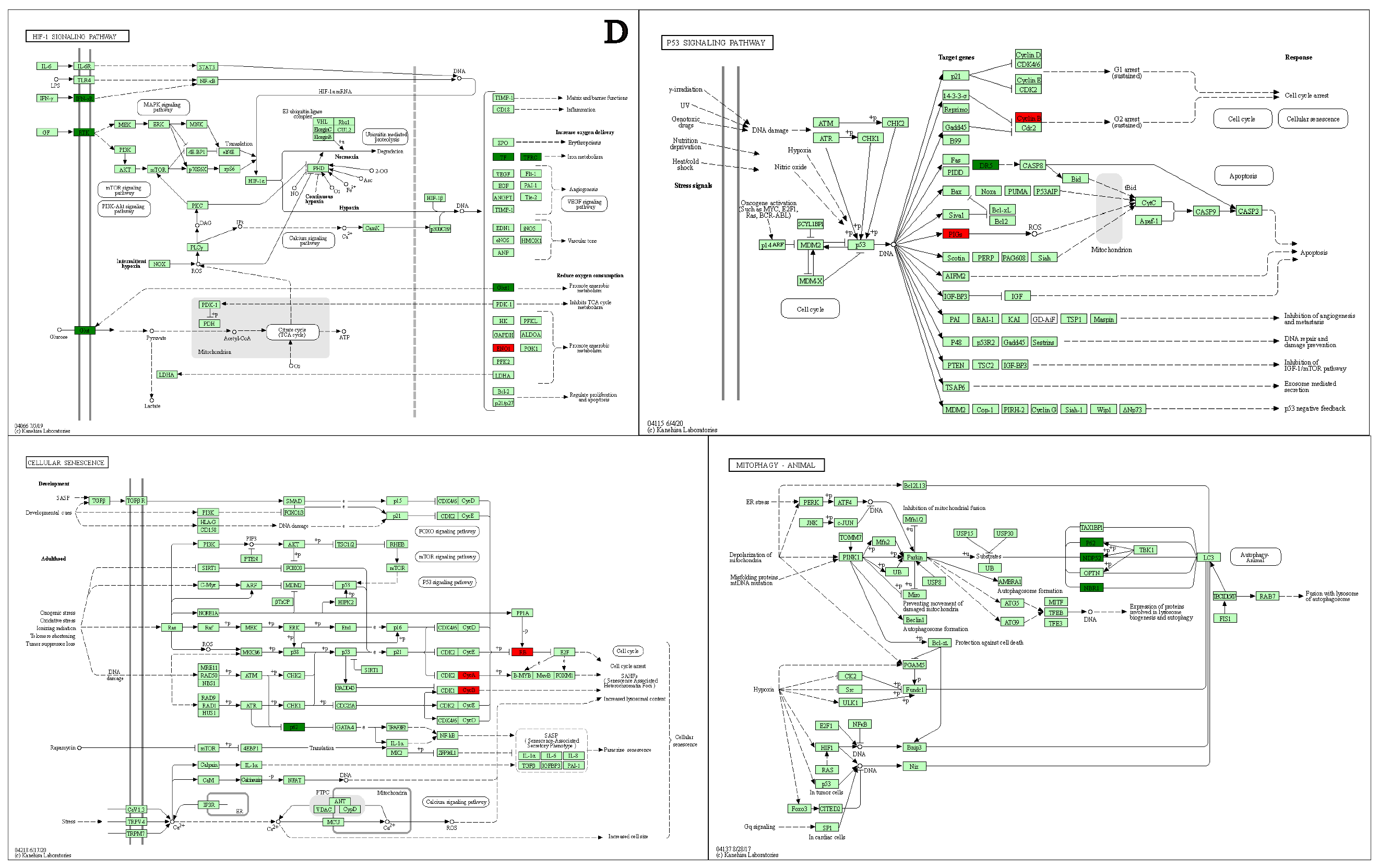


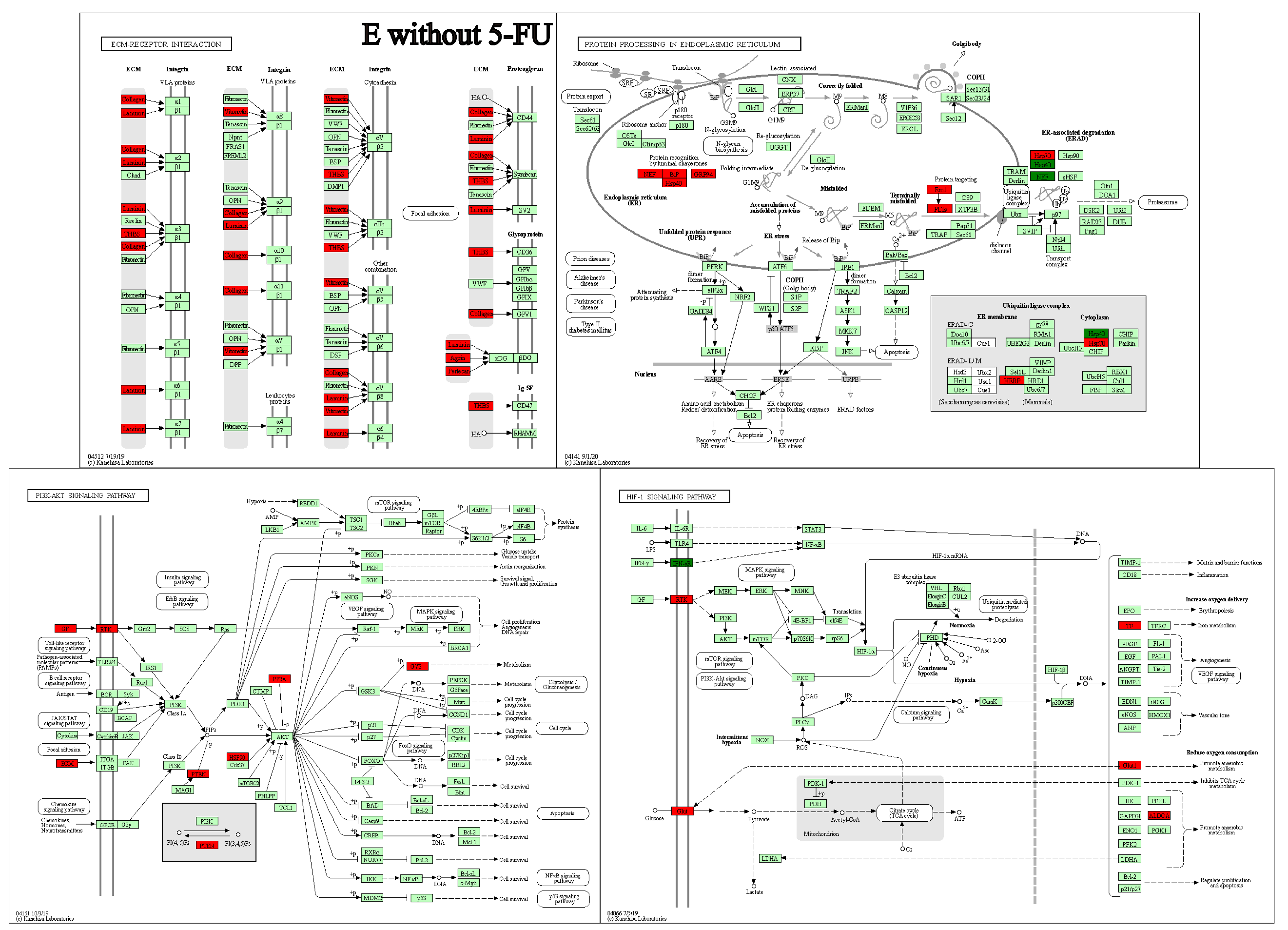


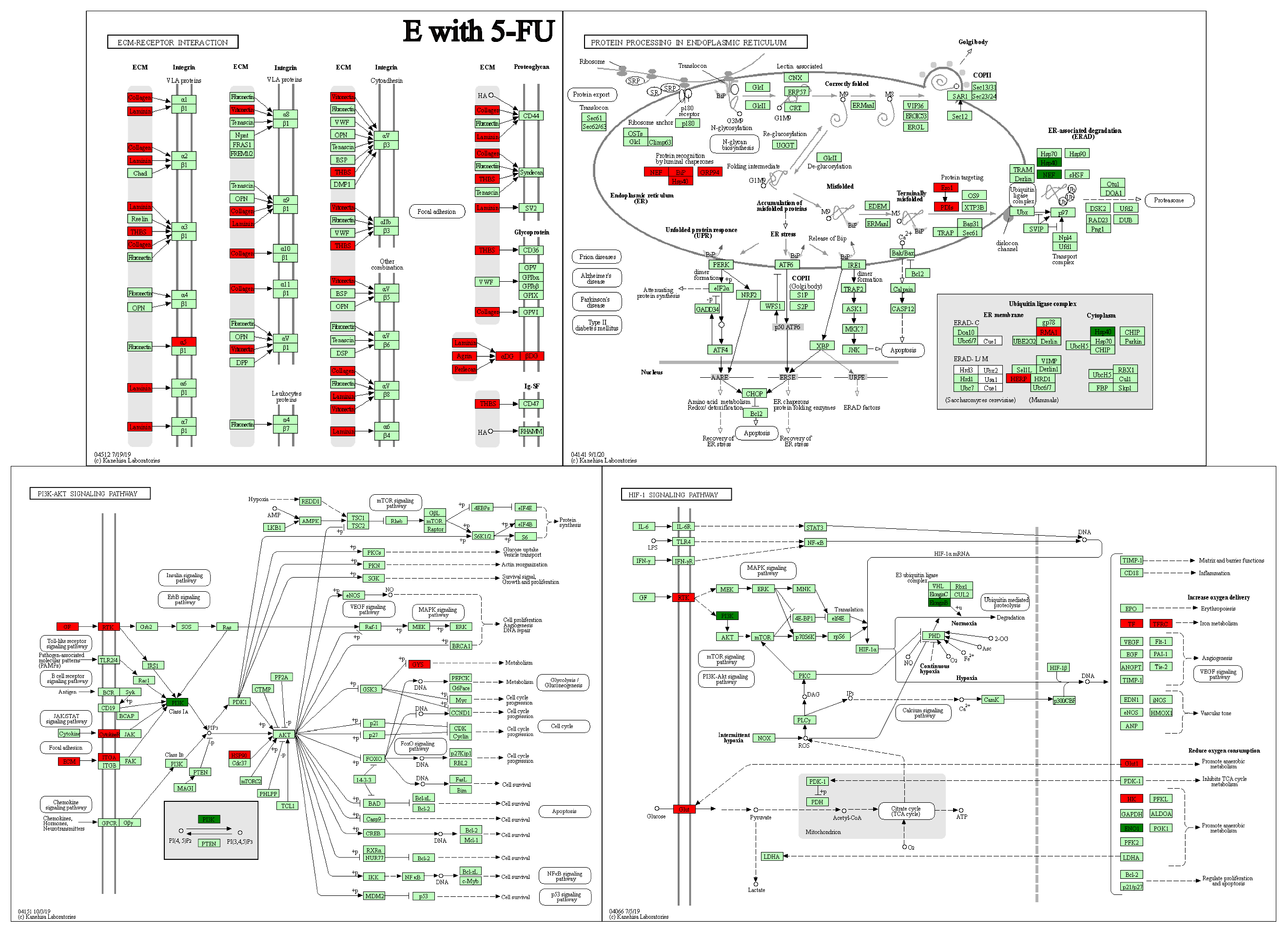


**Fig. S5**. (A) PCA analysis of proteome. (B) Heatmap of differential proteins between the control condition and 5-FU treatment. (C) The KEGG pathways involving proteome difference between the control condition and 5-FU treatment (p-value < 0.05, top 20 sorted according to the -log10 p-value). (D) KEGG map of differential proteins for 2D monolayer between the control condition and 5-FU treatment. (E) KEGG map of differential proteins between 2D monolayer and 3D MTSs before and after 5-FU treatment. The increase and decrease of proteins are marked with red and green rectangles, respectively.


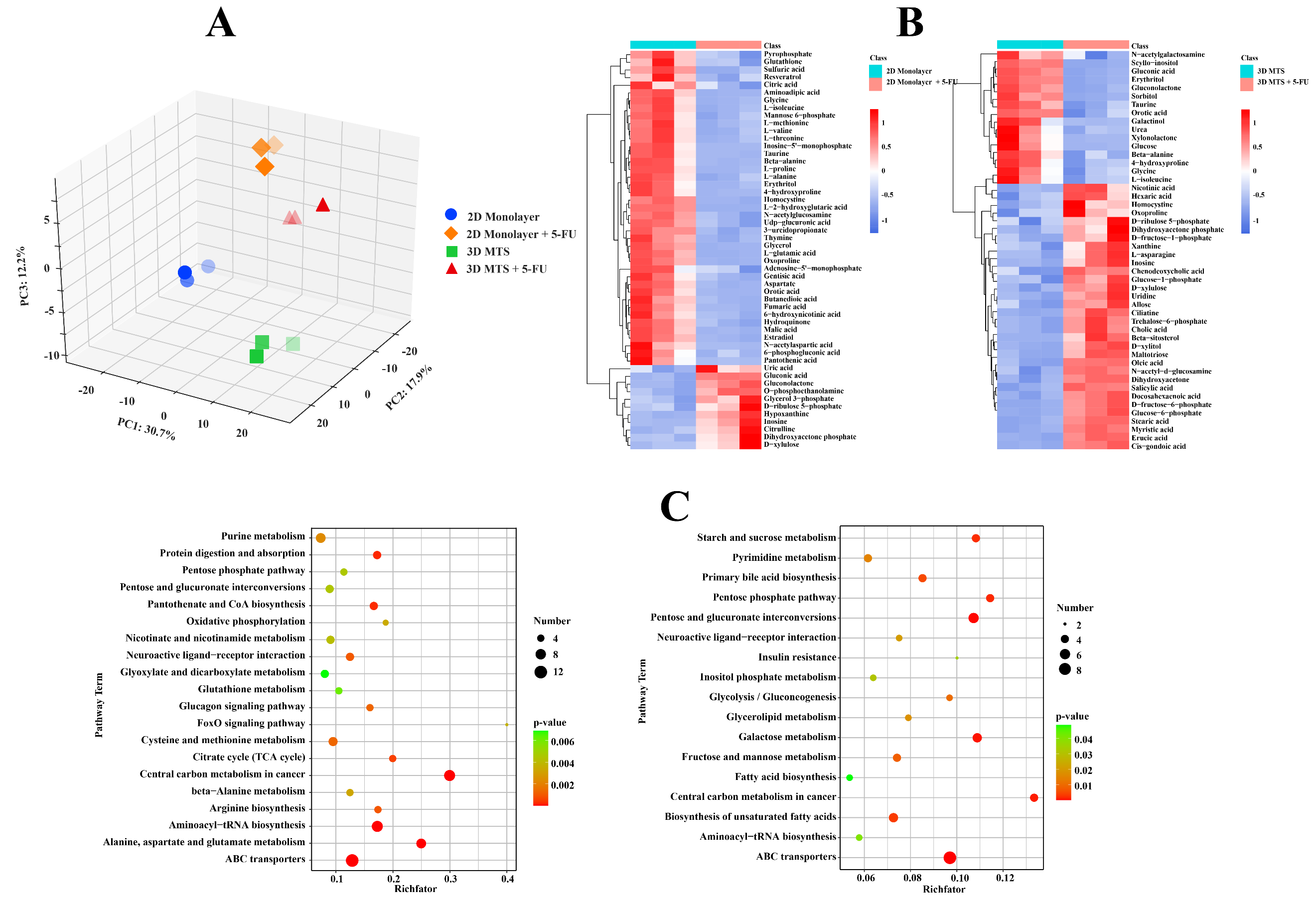


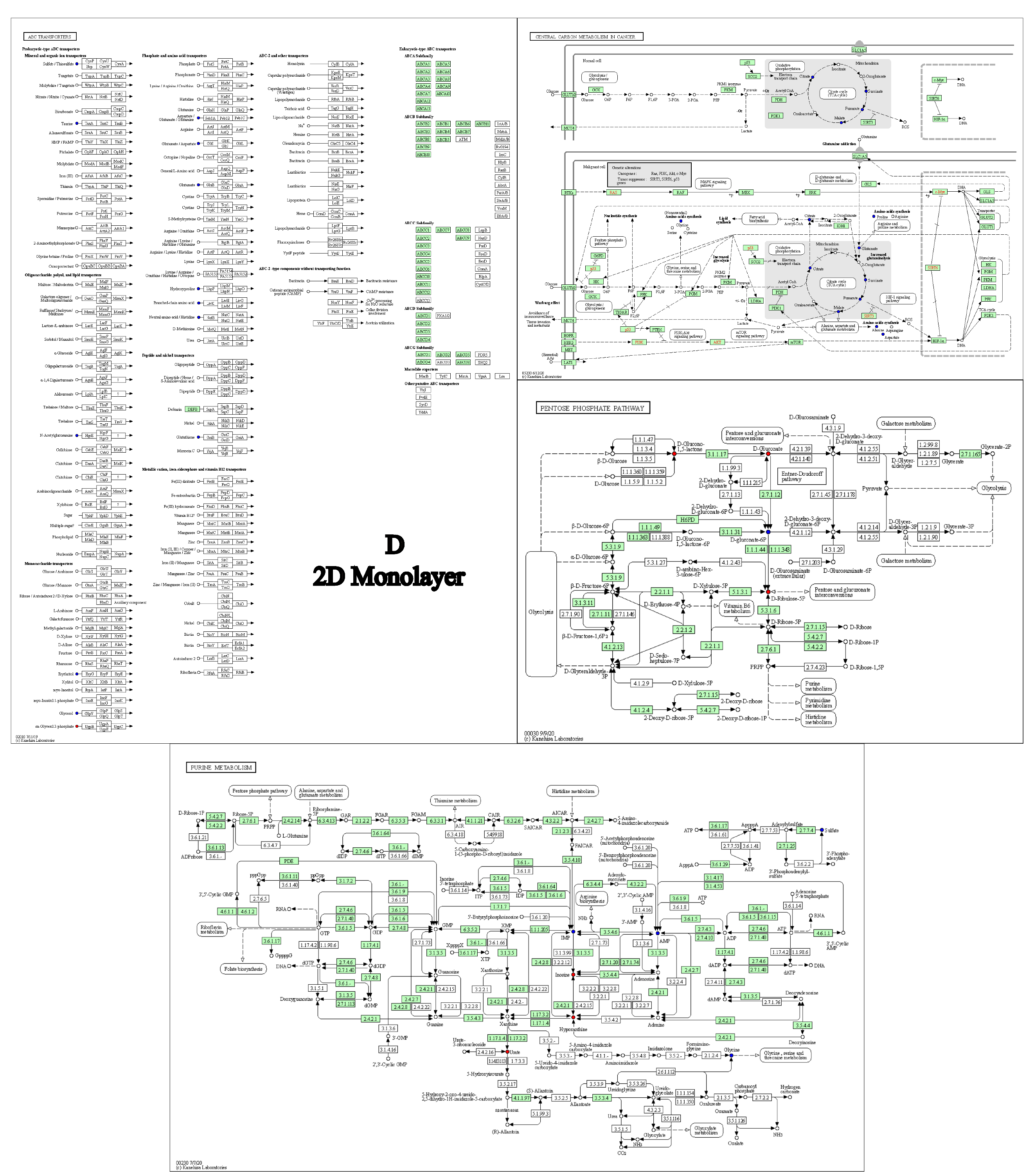


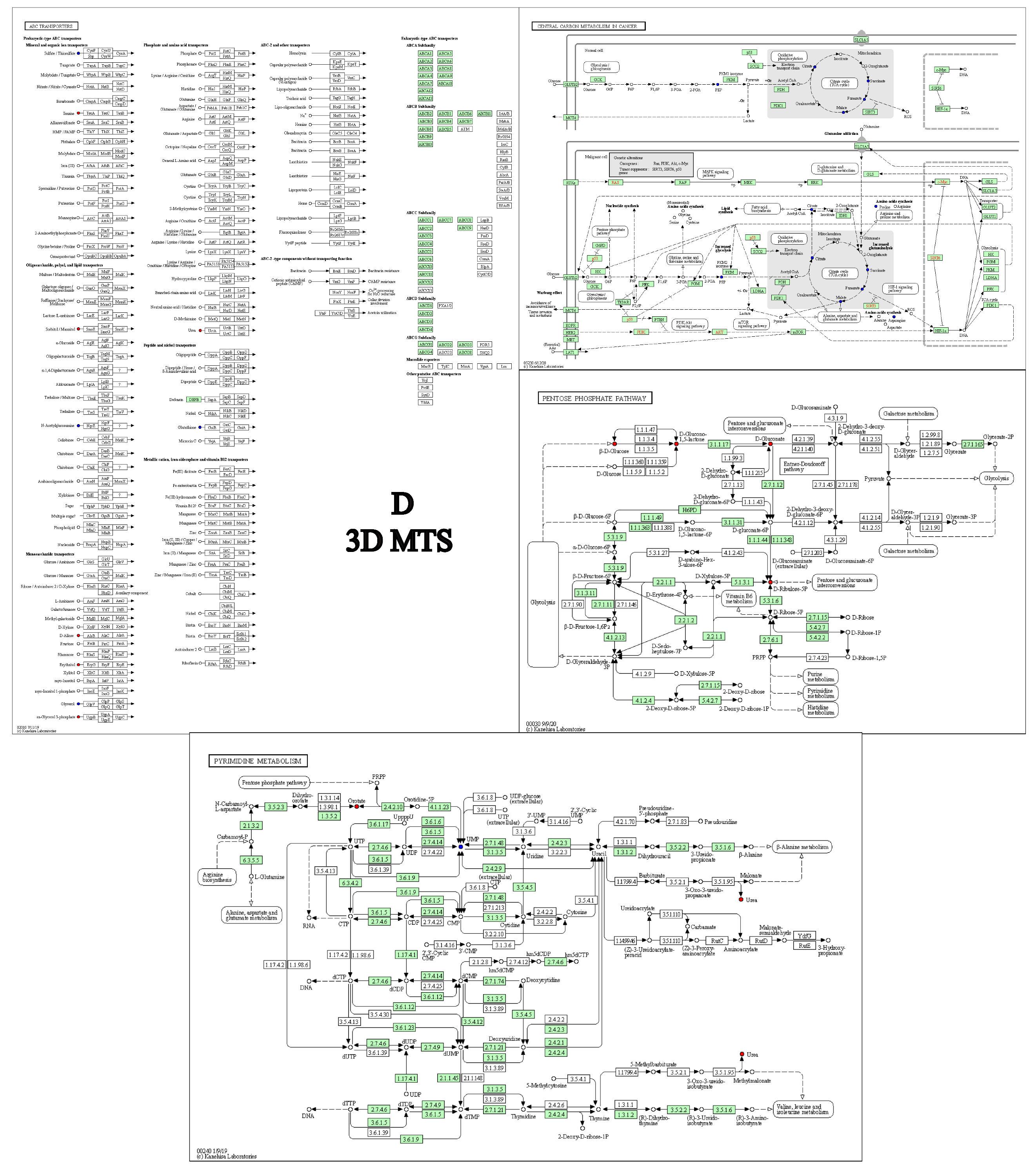


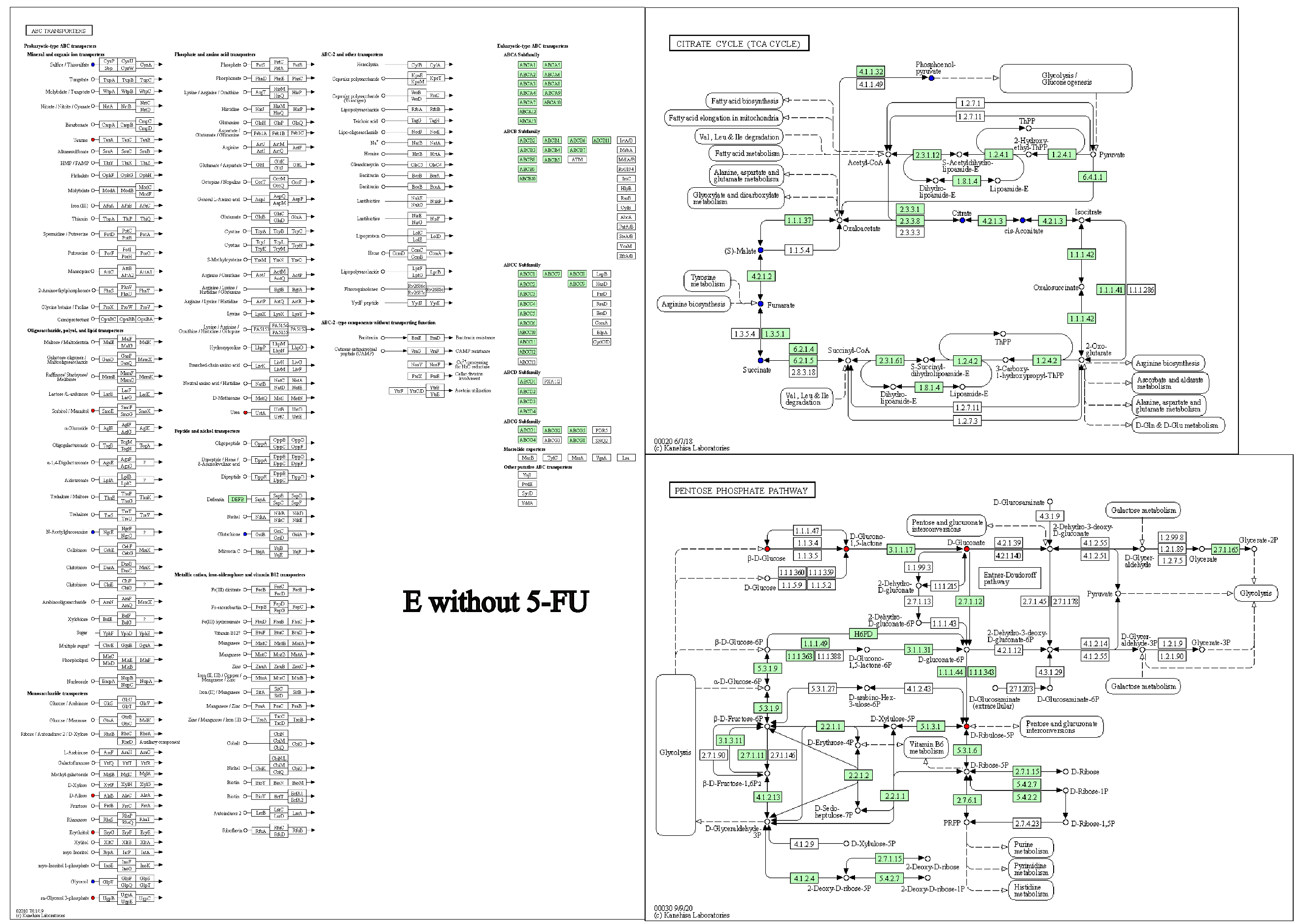


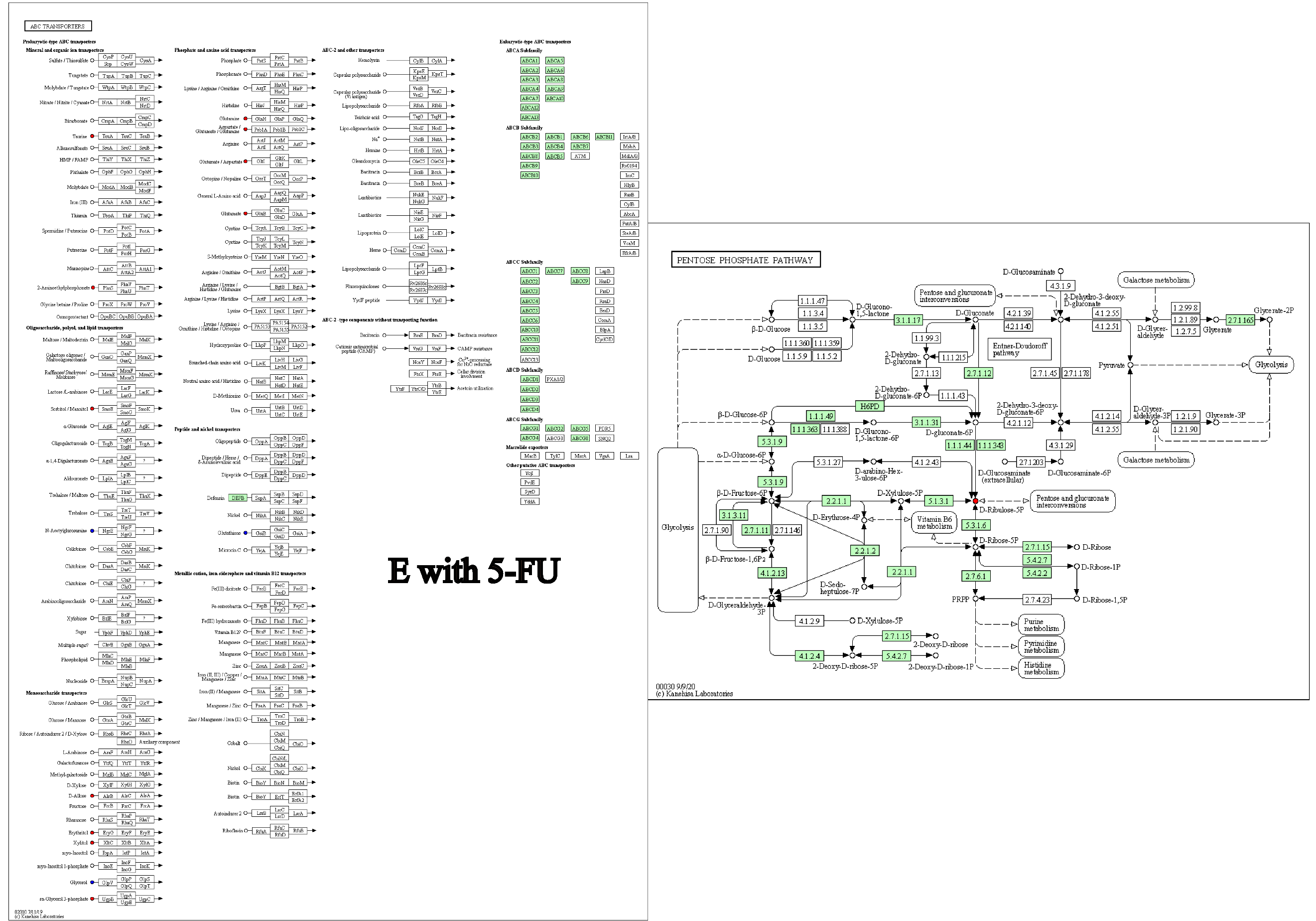


**Fig. S6**. (A) PCA analysis of metabolome. (B) Heatmap of differential metabolites between the control condition and 5-FU treatment conditions. (C) The KEGG pathways involving metabolome difference between the control condition and 5-FU treatment (p-value < 0.05, top 20 sorted according to the -log10 p-value). (D) KEGG map of differential metabolites for 2D monolayer and 3D MTS between the control condition and 5-FU treatment. (E) KEGG map of differential metabolites between 2D monolayer and 3D MTSs before and after 5-FU treatment. The increase and decrease of metabolites are marked with red and blue circles, respectively

**Supplementary tables**

**Table S1.** Primer sequences in this study.

| **Primer name** | **Primer sequence** | **Product length (bp)** |
| --- | --- | --- |
| *GLUT1-F*  *GLUT1-R* | CTGGCATCAACGCTGTCTTC  AACAGCGACACGACAGTGAA | 124 |
| *LDHA-F*  *LDHA-R* | AGCTGTTCCACTTAAGGCCC  TGGAACCAAAAGGAATCGGGA | 129 |
| *PFK1-F*  *PFK1-R* | TGCCCAAGGTATGAATGCTG  ACGCTTCACCAGGTTGTAGG | 247 |
| *DYPD-F*  *DYPD-R* | ACTCTGTGTTCCACTTCGGC  CAGGCATCTCATTGCTTCTCG | 162 |
| *TYMS-F*  *TYMS-R* | GTTGCTGTGGTTTATCAAGGGAT  TGGTCAACTCCCTGTCCTGA | 219 |
| *LAPTM4B-F*  *LAPTM4B-R* | GGAGCGATGAAGATGGTCGC  ATGCACATGTTGGCATCATCC | 227 |
| *BCL2-F*  *BCL2-R* | TCATGTGTGTGGAGAGCGTC  TCCACAAAGGCATCCCAGC | 133 |
| *BAX-F*  *BAX-R* | AACATGGAGCTGCAGAGGATG  GGGACATCAGTCGCTTCAGT | 298 |
| *MTOR-F*  *MTOR-R* | ATCTTGGCCATAGCTAGCCTC  ACAACTGGGTCATTGGAGGG | 107 |
| *VEGFA-F*  *VEGFA-R* | GCAGCTTGAGTTAAACGAACG  GGTTCCCGAAACCCTGAG | 94 |
| *TGFB1-F*  *TGFB1-R* | ACGTGGAGCTGTACCAGAAAT  TGAACCCGTTGATGTCCACT | 219 |
| *MET-F*  *MET-R* | TGGGCACCGAAAGATAAACCT  TCTCGGACTTTGCTAGTGCC | 130 |
| *CD44-F*  *CD44-R* | CCCTGCTACCAATAGGAATGATGT  GCTTTCCTTCGTGTGTGGGT | 115 |
| *ITGB1-F*  *ITGB1-R* | CCGCGCGGAAAAGATGAAT  ATGTCATCTGGAGGGCAACC | 252 |
| *FN1-F*  *FN1-R* | TTGCTCCTGCACATGCTTTG  TCGGGAATCTTCTCTGTCAGC | 208 |
| *LAMB1-F*  *LAMB1-R* | CGGAAAGGAAGACGGGAAGAA  TGCACAGGGCTAAGAAACTGAA | 135 |
| *HIF1A-F*  *HIF1A-R* | TTTTGGCAGCAACGACACAG  GTGCAGGGTCAGCACTACTT | 173 |
| *MYC-F*  *MYC-R* | GTAGTGGAAAACCAGCAGCCTC  AATACGGCTGCACCGAGTC | 97 |
| *ABCB1-F*  *ABCB1-R* | CAGAGGGGATGGTCAGTGTT  CCTGACTCACCACACCAATG | 87 |
| *ABCG2-F*  *ABCG2-R* | AAACCTGGTCTCAACGCCATC  TTCTCTCACCGTCAGAGTGCC | 201 |
| *VIM-F*  *VIM-R* | GGACCAGCTAACCAACGACA  GCAGCTCCTGGATTTCCTCT | 254 |
| *ACTB-F*  *ACTB-R* | CTGGAACGGTGAAGGTGACA  AAGGGACTTCCTGTAACAACGCA | 140 |

**Table S2**. Screening results of MTSs culture conditions.

| **Conditions** | **Results** | **Standards** |
| --- | --- | --- |
| Equipment | 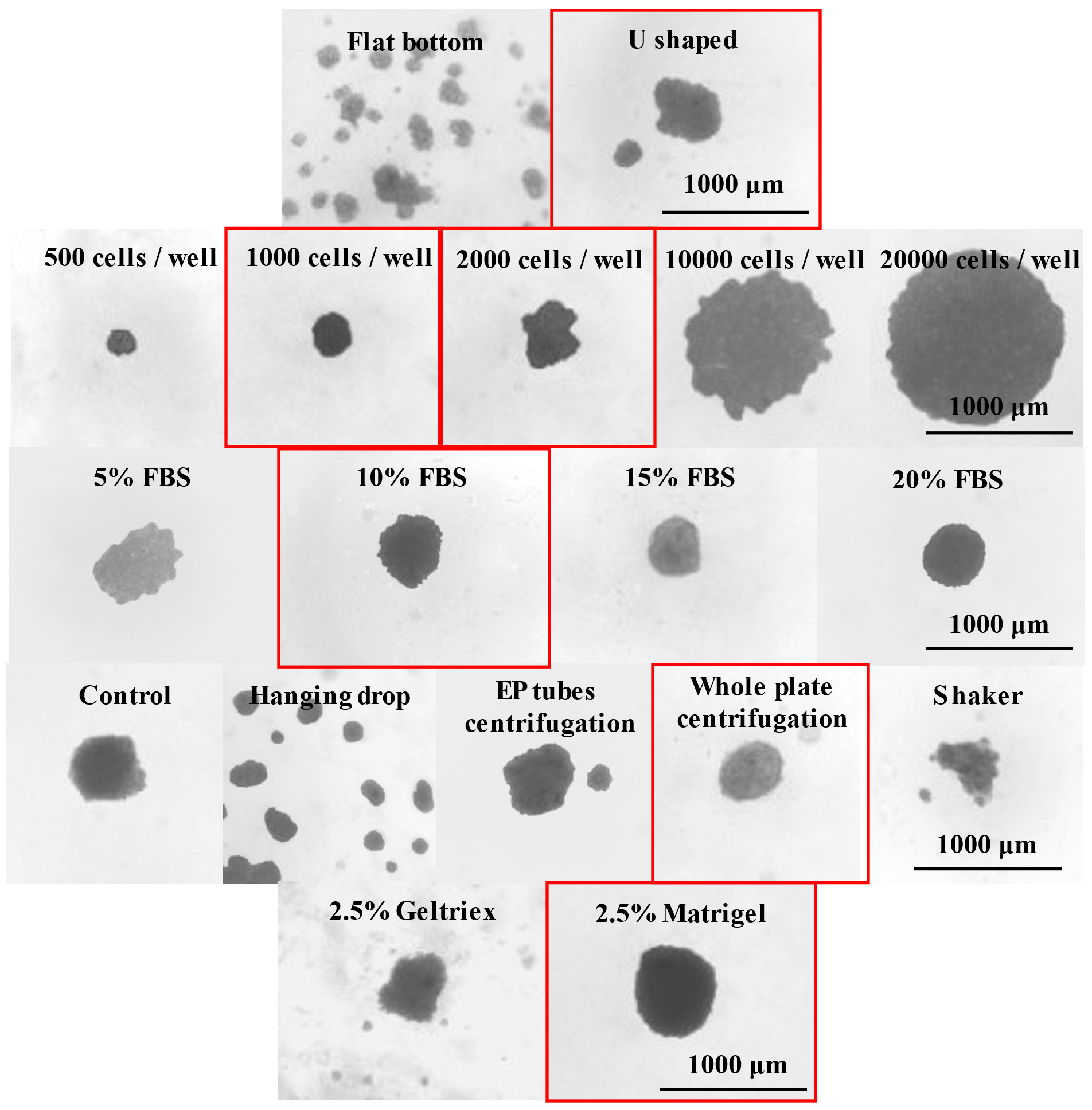 | Degree of aggregation |
| Density |  | 500 μm in diameter |
| Serum conc. |  | Tightness and cost |
| Treatment |  | Operation, MTS growth, repeatability |
| Supplement |  | Roundness, tightness, MTS growth |

**Table S3.** GO enrichment results between 2D monolayer culture and 3D MTS under the control condition.

| **Biological process** | **Cellular component** | **Molecular function** | **Impact** |
| --- | --- | --- | --- |
| Extracellular matrix organization | Extracellular exosome | Calcium ion binding | Up |
| Cellular protein metabolic process | Extracellular region | Extracellular matrix structural constituent | Up |
| Platelet degranulation | Collagen-containing extracellular matrix | Integrin binding | Up |
| Collagen fibril organization | Endoplasmic reticulum lumen | Chaperone binding | Up |
| Tissue development | Basement membrane | Extracellular matrix structural constituent  conferring tensile strength | Up |
| Retinoid metabolic process | Platelet alpha granule lumen | Heparan sulfate proteoglycan binding | Up |
| Blood vessel development | Tertiary granule lumen | Protein disulfide isomerase activity | Up |
| Negative regulation of amyloid fibril formation | Laminin-1 complex | Clathrin heavy chain binding | Up |
| Protein folding in endoplasmic reticulum | Laminin-10 complex | Propionyl-CoA carboxylase activity | Up |
| Collagen-activated tyrosine kinase  receptor signaling pathway | Laminin-11 complex | CoA carboxylase activity | Up |
| Mitochondrial translational elongation | Mitochondrion | RNA binding | Down |
| Mitochondrial translational termination | Mitochondrial inner membrane | Structural constituent of ribosome | Down |
| Mitochondrial translation | Mitochondrial matrix | Ubiquitin protein ligase binding | Down |
| Mitochondrial electron transport, NADH to ubiquinone | Mitochondrial small ribosomal subunit | Chaperone binding | Down |
| Response to unfolded protein | Mitochondrial nucleoid | NADH dehydrogenase (ubiquinone) activity | Down |
| Chaperone cofactor-dependent protein refolding | Mitochondrial large ribosomal subunit | Muscle alpha-actinin binding | Down |
| Mitochondrial transcription | Mitochondrial respiratory chain complex I | rRNA binding | Down |
| Negative regulation of mitochondrial RNA catabolic process | Glutamyl-tRNA (Gln) amidotransferase complex | Glutaminyl-tRNA synthase  (Glutamine-hydrolyzing) activity | Down |
| Negative regulation of chaperone-mediated protein folding | Mitochondrial intermembrane space  protein transporter complex | Mitochondrial ribosome binding | Down |
| Glutaminyl-tRNAGln biosynthesis via transamidation | TIM23 mitochondrial import inner  membrane translocase complex | Protein transporter activity | Down |

**Table S4.** GO enrichment results between 2D monolayer culture and 3D MTS under 5-FU treatment condition.

| **Biological Process** | **Cellular Component** | **Molecular Function** | **Impact** |
| --- | --- | --- | --- |
| Extracellular matrix structural constituent | Extracellular exosome | Calcium ion binding | Up |
| Cell adhesion | Extracellular region | Extracellular matrix structural constituent  conferring compression resistance | Up |
| Cellular protein metabolic process | Endoplasmic reticulum | Integrin binding | Up |
| Post-translational protein modification | Endoplasmic reticulum lumen | Iron ion binding | Up |
| Platelet degranulation | Collagen-containing Extracellular matrix | Heparin binding | Up |
| Receptor-mediated endocytosis | Basement membrane | Chaperone binding | Up |
| Tissue development | Extracellular matrix | Laminin binding | Up |
| Collagen fibril organization | Laminin-1 complex | Extracellular matrix structural constituent  conferring tensile strength | Up |
| Transport across blood-brain barrier | Laminin-11 complex | Heparan sulfate proteoglycan binding | Up |
| Negative regulation of amyloid fibril formation | Laminin-10 complex | Low-density lipoprotein  particle receptor binding | Up |
| Cell division | Mitochondrion | Identical protein binding | Down |
| Chaperone cofactor-dependent protein refolding | Extracellular exosome | ATP binding | Down |
| Cell redox homeostasis | Mitochondrial matrix | RNA binding | Down |
| Mitochondrial translational elongation | Mitochondrial inner membrane | Protein homodimerization activity | Down |
| Mitochondrial translational termination | Cytoskeleton | Ubiquitin protein ligase binding | Down |
| Mitochondrial translation | Secretory granule lumen | Enzyme binding | Down |
| Glycerol ether metabolic process | Ficolin-1-rich granule lumen | Caspase binding | Down |
| Synaptic vesicle uncoating | Mitochondrial small ribosomal subunit | CARD domain binding | Down |
| Regulation of clathrin-dependent endocytosis | Mitochondrial nucleoid | Protein disulfide oxidoreductase activity | Down |
| Nucleoside triphosphate biosynthetic process | Mitochondrial large ribosomal subunit | Ubiquitin-like protein ligase binding | Down |
